# Did crop domestication change the fitness landscape of root response to soil mechanical impedance? An *in silico* analysis

**DOI:** 10.1093/aob/mcae201

**Published:** 2024-11-27

**Authors:** Harini Rangarajan, Jonathan P Lynch

**Affiliations:** Department of Plant Science, The Pennsylvania State University, University Park, PA 16802, USA; Department of Plant Science, The Pennsylvania State University, University Park, PA 16802, USA

## Abstract

**Background and Aims:**

Root axes with greater penetration ability are often considered to be beneficial in hard soils. We hypothesized that maize root phenotypes with greater plasticity (meaning reduced elongation in response to mechanical impedance, i.e. a ‘stop signal’) have fitness advantages over phenotypes with reduced plasticity (i.e. unimpeded root elongation) in native (virgin, uncultivated) soils, by reallocating root foraging to softer, presumably wetter, soil domains, and that the value of the stop signal reduced with soil cultivation and crop domestication.

**Methods:**

We used *OpenSimRoot* to simulate native and cultivated soils and evaluated maize root phenotypes with varying axial and lateral root penetration ability in water, nitrogen (N) and impedance regimes associated with Neolithic agriculture.

**Key Results:**

The stop signal was advantageous in native soils but was less beneficial in cultivated, irrigated soils. Reduced root foraging in hard, dry topsoil enabled root growth in deeper domains where water is available, resulting in an improved balance of resource expenditure and acquisition. The value of the stop signal declined during crop domestication with the advent of irrigation, which increased water availability in the topsoil. Soil cultivation reduced N availability, while irrigation increased N leaching, resulting in a shift in the fitness landscape, with greater lateral root length (i.e. reduced plasticity) being advantageous by colocalizing root foraging with N availability. The importance of the stop signal is evident in modern high-input systems in which drought is a limiting factor.

**Conclusions:**

Our results support the hypotheses that the reduction of lateral root growth by mechanical impedance is adaptive in native soil, but became less adaptive with soil cultivation and irrigation associated with Neolithic agriculture.

**Significance statement**

Roots stop elongating when they encounter hard soil. Simulations of ancient soils indicate that this response is useful in native soil but became less useful during crop domestication with the advent of irrigation and cultivation in Neolithic agriculture.

## INTRODUCTION

Reduced crop yield due to mechanical impedance is a global problem (Lal, 1985; Correa *et al.*, 2019; Bengough *et al.*, 2011; Lynch, 2018). In low-input agroecosystems characteristic of developing nations, crop productivity is limited by drought and low soil fertility, which are being exacerbated by ongoing soil degradation and climate change (St. Clair and Lynch, 2010; Lynch, 2018). In high-input agroecosystems, loss of soil organic matter, compaction and degradation of soil structure caused by conventional mechanized tillage have increased soil mechanical impedance (Hamza and Anderson, 2005; Batey, 2009; Keller *et al.*, 2019; Lynch *et al.*, 2022). Yield losses from compaction are greater when soil is affected by another constraint such as drought, since dry soils have much greater soil impedance than moist soils (Whiteley and Dexter, 1982; Bengough *et al.*, 2011; Colombi *et al.*, 2018; Lynch, 2022*a*). The detrimental effect of mechanical impedance on root growth results in limited soil exploration leading to reduced capture of soil resources. Root adaptations that permit better penetration of hard soils are generally believed to be beneficial (Whalley *et al.*, 2008; Whitmore and Whalley, 2009; Chimungu *et al.*, 2015; Lynch and Wojciechowski, 2015; Chen *et al.*, 2022). Ethylene is involved in several processes that occur when roots confront hard soils (Kays *et al.*, 1974; Sarquis *et al.*, 1991; He *et al.*, 1996; Pandey *et al.*, 2021; Schneider *et al.*, 2021). Diffusion of ethylene is reduced in soils with reduced porosity due to compaction or flooding, resulting in an accumulation near root tissue thereby triggering a signalling cascade which stops root growth (Pandey *et al.*, 2021). Formation of multiseriate cortical sclerenchyma, which stiffens roots and improves penetration of hard soil, is influenced by ethylene (Lynch *et al.*, 2022). Roots commonly respond to hard soil by root thickening (Kays *et al.*, 1974; Materechera *et al.*, 1991) which is thought to be advantageous by reducing mechanical stress (Hettiaratchi, 1990; Materechera *et al.*, 1991; Sarquis *et al.*, 1991; Kirby and Bengough, 2002). However, greater root diameter is associated with reduced elongation rates and greater metabolic cost of root elongation (Bengough and Mullins, 1991; Croser *et al.*, 2000; Colombi *et al.*, 2019; Vanhees *et al.*, 2020). Roots with less radial expansion in response to mechanical impedance had better penetration ability (Vanhees *et al.*, 2020, 2022). Genotypes that thicken in response to hard soil are less able to cross a hard soil layer than those whose anatomical phenotype remains unchanged, mediated by ethylene which acts as a stop signal for root growth (Vanhees *et al.*, 2020, 2022). Although research on root responses to mechanical impedance generally assumes that the ability to penetrate hard soil is advantageous in terms of soil resource capture, it has been proposed that the stop signal-mediated plasticity of root growth in response to mechanical impedance is adaptive by reallocating root foraging to softer, presumably wetter, soil domains (Lynch *et al.*, 2022). Root plasticity in response to mechanical impedance may be advantageous by preventing root foraging into soil domains that are hard because of, for example, surface drying (Lynch *et al.*, 2022).

Intraspecific variation in root penetration ability exists in several crops including maize, cotton, rice, wheat and common bean, suggesting that the adaptive value of root phenotypes for enhanced penetration in hard soils is variable (Cairns *et al.*, 2004; Botwright Acuña *et al.*, 2007; Bushamuka and Zobel, 1998; May and Kasperbauer, 1999; Kubo *et al.*, 2006; Grzesiak *et al.*, 2014; Chimungu *et al.*, 2015; Colombi and Walter, 2015; Rivera *et al.*, 2019; Vanhees *et al.*, 2020; Schneider *et al.*, 2021). Root class (i.e. primary, seminal, axial, lateral, nodal, etc.) specific variation in penetration ability has significant effects on the spatiotemporal distribution of root foraging and resource acquisition (Strock *et al.*, 2022). Wild crop ancestors and landraces differ in the expression of various root phenotypes (Burton *et al.*, 2013; Perkins and Lynch, 2021; Schneider *et al.*, 2021; Lopez-Valdivia *et al.*, 2022; Massas *et al.*, 2022). Multiseriate cortical sclerenchyma, which enhances penetration of hard soil, is found in cultivated crop taxa and early maize domesticates but not in their wild ancestors (Schneider *et al.*, 2021; Lopez-Valdivia *et al.*, 2022), suggesting that the fitness landscape for root adaptations to mechanical impedance has changed through crop domestication and evolution.

Cultivated agriculture (in this context signifying the husbandry of desired plants, including soil tillage by any means such as manual and animal-drawn tillage, planting, weed control, protection from herbivores, etc.) arose in fertile alluvial plains with irrigation (Lal *et al.*, 2007; Riehl *et al.*, 2012; Sandor and Homburg, 2017). Irrigation makes the soil climate more humid (Yaalon and Yaron, 1966). There is evidence for cultivation of crops under irrigation as early as 10 000 years BP (Dillehay *et al.*, 2007; Araus *et al.*, 2014; Neely *et al.*, 2022). Soil cultivation reduced soil fertility resulting in increasing N limitation in Neolithic agriculture (Araus *et al.*, 2014). Crop domestication and associated anthropogenic changes in soil environments brought about by irrigation and cultivation may have influenced the evolution of root architecture and anatomy (Lopez-Valdivia *et al.*, 2022). It has been proposed that the advent of irrigation and soil cultivation in Neolithic agriculture transformed the fitness landscape and selection pressures associated with root responses to soil mechanical impedance (Lynch *et al.*, 2022). Crop ancestors and landraces were selected with multiple stresses, competition, significant root loss and heterogeneous resource distribution, which favoured plasticity in response to resource availability (Lynch, 2018). However, in modern high-input agroecosystems human management has removed many of these constraints to root function (Lynch, 2018; Schneider and Lynch, 2020; Lynch *et al*, 2022). In the context of soil mechanical impedance, growth plasticity in native soil (i.e. soil in its native state, prior to human intervention, unmodified by agriculture) may have permitted plants to focus root foraging effort on softer, deeper, wetter soil domains. The advent of cultivation and irrigation with Neolithic agriculture would have reduced water limitation but also would have decreased soil fertility and increased N leaching, in which case roots with greater ability to penetrate hard soil would be advantageous in order to attain greater rooting depth to acquire N (Lynch *et al.*, 2022). Climate change and shifts from conventional mechanical tillage towards conservation agriculture are returning to cultivated soils some characteristics of native soil, which could alter the adaptive value of the stop signal (Lynch *et al.*, 2022).

To test the hypothesis that the utility of the stop signal in response to soil impedance has changed during crop domestication and evolution, we evaluated root phenotypes with varying root growth plasticity in a range of ancient and modern soil environments *in silico*. We employed *OpenSimRoot_v2*, an open-source functional–structural plant/soil model with the ability to simulate the effects of water and nutrient limitation on plants and soils as well as the ability to simulate the spatiotemporal dynamics of soil mechanical impedance and its effects on root growth and resource capture in drying soils (Strock *et al.*, 2022; Schäfer *et al.*, 2022).

## METHODS

*OpenSimRoot_v2* was used (Schäfer *et al.*, 2022) in this study to simulate plant and soil responses to drought. *OpenSimRoot_v2*, like its predecessor *OpenSimRoot*, is a dynamic open-source functional–structural plant/soil model which explicitly simulates root growth and soil in three dimensions and accounts for interactions between soil properties, root growth, the metabolic costs of root construction and maintenance, and water and nutrient acquisition at the scale of individual root segments over time, with added functionality.

### Plant parameters

In *OpenSimRoot*, root system architecture is discretized into small (~1 cm) root segments modelled as connected cylinders or truncated cones in three dimensions (Postma *et al.*, 2017). Nutrient uptake by the root system is estimated by integrating the nutrient uptake over all root segments. Carbon required for root growth is derived initially from seed reserves, and leaf photosynthesis becomes the dominant source of carbon after the seedling has been established. The shoot is simulated through a number of state variables and is not simulated geometrically. *OpenSimRoot_v2* was used in this study; *OpenSimRoot_v2* differs from *OpenSimRoot* in the strength of its ability to simulate photosynthetic processes as well as both plant and soil responses to water limitation. *OpenSimRoot_v2* includes the Farquhar–von Caemmerer Berry (FvCB) model for photosynthesis, leaf gas exchange and stomatal conductance, leaf temperature and energy balance models, sun/shade model for leaf-to-canopy scaling, a model for nitrogen-limited photosynthesis, water stress response functions and models for simulating day–night cycles and the corresponding carbon allocation. Carbon is partitioned among roots, shoots and leaves according to potential growth rates limited by stress and available resources. The strength of carbon sinks is based on potential growth rates of different organs. Carbon required for root maintenance (respiration, root exudation) is prioritized over growth sinks. Shoot is prioritized over root for carbon partitioning and, among roots, major axis has priority over fine roots.

The soil domain is simulated by a finite element model which contains nodal values for water content, nutrient content and several soil properties such as bulk density. A soil impedance module (Strock *et al.*, 2022) was included to simulate the dynamic 3D effects of soil texture, bulk density, water status and root water capture on soil penetration resistance. In the impedance module, soil strength is represented as impedance to linear penetration and scales linear extension and diameter growth rates of each root through functions which relate soil strength to soil water status and soil bulk density and a function which defines root elongation rate as limited by soil physical conditions (Strock *et al.*, 2022). Mass flow and diffusion of phosphorus in the rhizosphere around the root is simulated using Barber–Cushman’s model (Itoh and Barber, 1983) while water flow, using Richard’s equation (Richards, 1931), and nitrate movement (using the convection dispersion equation) in the soil domain are simulated using SWMS_3D (Šimůnek *et al*, 1995; Somma *et al.*, 1998). Uptake is simulated using a Michaelis–Menten kinetics formulation (Barber, 1995). Organic matter mineralization follows the Yang and Janssen model (Yang and Janssen, 2000). Ammoniacal nitrogen is not modelled, as nitrification is generally rapid in the field conditions being simulated (Barber, 1995). By using *OpenSimRoot_v2* with the impedance module, we can capture the effects of water deficit on plants through direct effects of water deficit on plant growth as well as through the effects of soil penetration resistance on root growth. Further information on *OpenSimRoot_v2* is provided by Schäfer *et al.* (2022).

In the impedance module, soil strength is computed from bulk density, water status and position in the soil column using the pedotransfer function from Gao *et al.* (2016). The effect of soil strength on linear extension and diameter growth rates is simulated by including a function that calculates the soil strength-dependent root impedance factor which defines the root elongation rate as limited by soil physical conditions. Bulk density is provided in the input files and all the other inputs are obtained from *OpenSimRoot*’s existing water module. Impedance at the precise time and place where root growth occurs is computed, thus allowing dynamic representation of the interactions between soil hardness and water content as they evolve through the growing period (Strock *et al.*, 2022).

### Root responses to penetration resistance

**Fig. 1.**
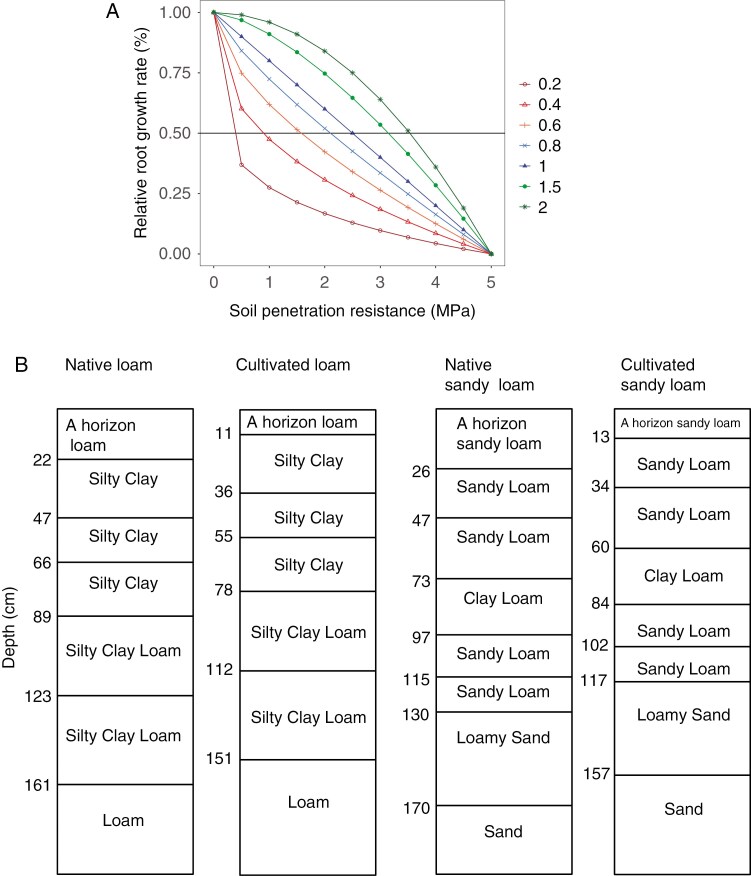
Relationship between relative root growth rate and soil penetration resistance (A). Loam and sandy loam soil profile used in the study (B). Cultivated soils were assumed to have a reduced A horizon (50 % of the native soil), reduced organic matter (50 % of native soil) and greater bulk density (10 % greater than native soil) in the A horizon. The parameters corresponding to the organic matter and bulk density are given in Table 2.

Soils are strong when they are compact or dry, reducing root elongation which in turn has detrimental effects on plant growth (Passioura, 2002; Bengough *et al.*, 2006). Root elongation is affected in soils with values of 0.8–2 MPa, and penetrometer pressures of 2–2.5 MPa are sufficient to significantly impede root elongation, with root elongation ceasing completely at a resistance of ~5 MPa (Bengough and Mullins, 1991; Bengough *et al.*, 2006; Whitmore and Whalley, 2009). To implement these conditions, we modified the function that relates soil mechanical impedance to root elongation from the function that is originally described in Strock *et al.* (2022) to the following function:


$$\begin{array}{l} y=1-{\left(x/5000\right)}^{a} \end{array}$$

where *y* is root growth rate reduction, *x* is soil mechanical impedance and *a* is a factor that determines the shape of the curve and thus determines the penetration ability of the root. A value of *a* = 1 corresponds to a linear reduction in root growth rate with increased soil mechanical impedance between 0 and 5 MPa; *a* < 1 results in faster reduction in root growth (less penetration ability) and thus renders the roots sensitive to increased mechanical impedance; and *a* > 1 results in slower reduction in root growth (greater penetration ability) and less sensitivity to increased soil mechanical impedance (Fig. 1A). By using this function, we can simulate root phenotypes that vary in their soil penetration ability (which is the inverse of the sensitivity of their ‘stop signal’), all of which cease to grow at 5 MPa. Forty-nine phenotypes corresponding to the factorial combination of seven levels each of axial and lateral root penetration ability were simulated by varying the value of *a* (0.2, 0.4, 0.6, 0.8, 1, 1.5, 2).

A Figshare repository (https://figshare.com/s/5fd66984b4fe27185582) contains all the input files and source code for the version of *OpenSimRoot* used in this study.

### Soil parameters

Inceptisols (corresponding to Cambisols in the FAO WRB nomenclature) are one of the major soil orders that comprise the paleosols of the Holocene (Holdridge and Leigh, 2018) and are representative of soils derived from alluvial sediments which sustained many early agricultural civilizations (e.g. McAuliffe *et al.*, 2001; Retallack, 2001; Jaradat and Timlin, 2022). Two inceptisols developed from alluvial deposits were selected (Markewich *et al.*, 1988), one with a sandy loam A horizon and one with a silt loam A horizon. The sandy loam soil had a surface sandy loam extending to 73 cm depth followed by clay loam horizon up to 97 cm, followed by sandy loam from 115 to 130 cm. The silt loam soil had a silty loam soil up to 22 cm followed by a silty clay soil up to a depth of 89 cm and a silty clay loam soil from 89 to 160 cm (Fig. 1B).

Maize root phenotypes varying in axial and lateral root penetration ability were tested in soil conditions representing a native soil, a soil under conditions representative of Neolithic agriculture with early crop domesticates, and modern agriculture with added inputs under well-watered and water-stressed conditions. The assumptions of native and cultivated soils considered in this study are given in Table 1. The soil texture, bulk density and organic matter parameters of the soil profile as obtained from the literature (Markewich *et al.*, 1988) without any modifications were considered representative of native soil. Several studies have documented loss in organic matter when virgin soils and adjacent cultivated soils are compared. Soil changes upon cultivation of virgin soils include accelerated erosion, topsoil compaction, losses of organic matter and nutrients including N and P. The depth of cultivation-induced changes was found to be limited to the upper soil layers with 9–15 % greater bulk density and 45 % less organic carbon in cultivated A horizons than in uncultivated soils (Sandor *et al.*, 1986). Comparisons of prehistorically cultivated soils to nearby uncultivated counterparts suggest decreased N and P fertility (Araus *et al.*, 2014). The cultivated soils were assumed to have lost 50 % of the A horizon and the remaining layer was considered to have 50 % of the organic matter as that of the native soil to represent soil erosion and loss of organic matter with cultivation (Fig. 1B; Table 1). Bulk density in the A horizon was assumed to increase by 10 % in the cultivated soils (Table 1). Soils used for Neolithic agriculture are potentially productive by modern agricultural standards. Nitrogen is the main limiting nutrient in cultivated, irrigated soil (Sandor and Gersper, 1988). Agricultural utilization of virgin soils marked a 20–50 % reduction of soil organic matter resulting in release of mineral N which can be taken up by plants (Reinhorn and Avnimelech, 1974).

**Table 1.** Assumptions of native and cultivated soils described in this study.

Native	Cultivated well-watered low N
Undisturbed soil	Area cleared, manual tillage, rainfall-induced topsoil erosion, harder topsoil due to loss of organic matter from erosion and manual cultivation Under irrigation Irrigation-induced leaching of available nitrate to deeper soil No exogeneous fertility inputs

**Table 2.** Environmental scenarios simulated in this study. The main text contrasts the two canonical soil regimes relevant to crop domestication, i.e. native (uncultivated) soil prone to water deficit stress, and cultivated soil prone to degradation and N stress, both at a Neolithic atmospheric CO_2_ concentration of 270 ppm. Scenarios provided in the Supplementary Data include native soil with adequate water and N, and cultivated soil either with combined water and N deficit stress or with adequate water and N, all at either current atmospheric CO_2_ (402.9 ppm) or a future elevated atmospheric CO_2_ concentration (500 ppm).

Native soil	Cultivated soil
**Main text**	**Main text**
270 ppm CO_2_ Water deficit stress	270 ppm CO_2_ Nitrogen deficit stress, soil degradation
**Supplementary Data**	**Supplementary Data**
402.9 and 500 ppm CO_2_ Unstressed	270, 402.9 and 500 ppm CO_2_ Combined nitrogen and water deficit stress Unstressed

In our simulations, N was available from mineralization while some was assumed to be readily available from previously mineralized organic matter in the native soil by including it in the parameterization of the initial N availability. In cultivated soils, the contribution from mineralization was parametrized to be very low due to low organic matter content. Plants are known to take up N in the form of dissolved organic N (Moreau *et al.*, 2019; Farzadfar *et al.*, 2021), but dissolved organic N was not simulated in our study. Two conditions corresponding to no water limitation and drought environments were implemented by changing the amount of precipitation, temperature and initial soil water content. The van Genuchten parameters and saturated hydraulic conductivity parameters were calculated based on the soil texture, bulk density and water content at field capacity and permanent wilting point. Water content corresponding to field capacity and permanent wilting point were estimated using the Saxton–Rawls function (Saxton and Rawls, 2006) using the soil texture, bulk density and organic matter data obtained from Markewich *et al.* (1988). The values of θ_r_ (residual water content) ranged from 0.0249 to 0.0911, θ_s_ (saturated water content) from 0.3881 to 0.5097, and α and *n* (the factors that shape of the soil-water retention curve) had values of 0.0057–0.0611 and 1.2432–1.5812 respectively. The bulk density and van Genuchten parameters used in this study for the two soils and the different agricultural systems are available in Supplementary Data Table S1.

Forty-nine phenotypes corresponding to seven levels of axial root penetration ability and seven levels of lateral root penetration ability were simulated in two soils, a loam and a sandy loam Inceptisol, under three management scenarios, native soil, cultivated soil with low N, and cultivated soil with high N under two levels of water availability, no water limitation and water limited. Water limitation was simulated by setting precipitation rates to zero after a certain time. Three levels of atmospheric CO_2_ representative of the conditions present during Neolithic agriculture (270 ppm), current conditions (402.9 ppm) and future CO_2_ (500 ppm) were included in the study. This resulted in 3528 simulations (49 phenotypes × 2 soils × 3 management scenario × 2 water levels × 3 CO_2_ levels conducted in duplicate).

## RESULTS

**Fig. 2.**
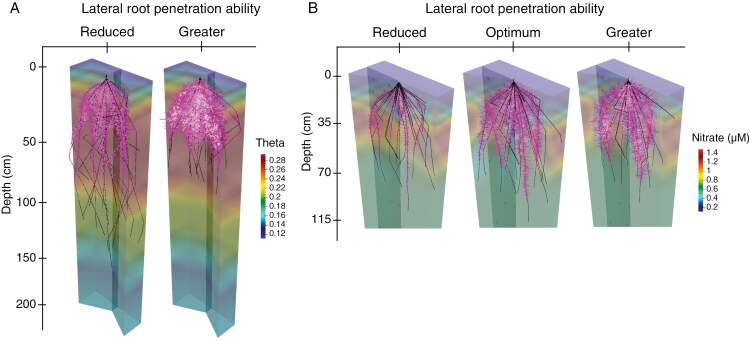
Representative maize root phenotypes at 40 d after germination in a loam Inceptisol as a native undisturbed soil with dry topsoil (A) and an irrigated cultivated soil with low N (B). The soil is coloured by volumetric water content in the native soil (A) and by nitrate concentration in the cultivated soil (B). Both nitrate and water availability were considered in the simulations. Native undisturbed soil with dry topsoil was limited in water, but nitrate was unlimited and so only volumetric water content is shown in the figure. Irrigated cultivated soil was limited only in nitrate and so only nitrate concentration is shown in the corresponding figure. The maize root phenotypes vary in their lateral root penetration ability. Phenotypes corresponding to reduced, optimal and greater lateral root penetration ability are represented in the figure. Note that the phenotype with reduced lateral root penetration ability is also the optimal lateral root penetration ability in the native soil.

A representative illustration of simulated maize root phenotypes varying in axial and lateral root penetration ability in a native and a cultivated irrigated loam Inceptisol is shown in Fig. 2. For brevity, the main focus of this section is the comparison of the two dominant soil regimes in the context of crop domestication, i.e. native soils subject to water stress in contrast to cultivated soils with irrigation but with reduced N availability. Other possible scenarios including native soils without water stress and cultivated soils with varying water and N availability are shown in the Supplementary Data (results for a loam soil texture and for the environmental scenarios of native soil without stress, cultivated soil without stress, cultivated soil with water deficit stress, and cultivated soil with both water and nitrogen deficit stress are shown in Supplementary Figs. S2–S15).

### Feedback of soil physical parameters on root–soil interaction determines resource capture

**Fig. 3.**
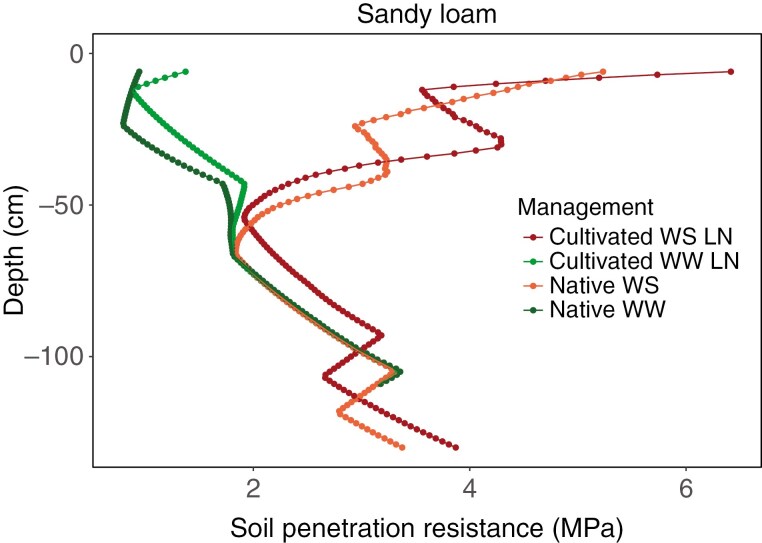
Penetration resistance profile in loam and sandy loam Inceptisols under different soil management conditions on day 40 of simulation. Native WS – native soil under water stress; Native WW – native soil with no water stress; Cultivated WS LN – cultivated soil with water limitation and low N availability; Cultivated WW LN – cultivated irrigated soil with low N availability; the cultivated soil with water limitation had similar penetration resistance under low N as well as high N availability.

The pattern of soil penetration resistance in a native soil and a cultivated soil under well-watered (WW) and water-stress (WS) conditions in two soil textures (sandy loam and loam) is represented in Fig. 3. The differences in the thickness and bulk density of the topsoil and organic matter content used to simulate the soil physical properties and water retention parameters of the native and cultivated soils resulted in differences in soil penetration resistance with depth. In both the loam and the sandy loam soil, penetration resistance was much lower in moist (WW) than in dry (WS) soil. Native soils had less soil penetration resistance in the topsoil compared to cultivated soils. A greater proportion of the total profile had soil penetration greater than 4 MPa in the loam than in the sandy loam under water deficit. The topsoil had greater soil strength than deeper soil under water deficit. Maize root phenotypes varying in axial and lateral root penetration ability were evaluated in these soils.

**Fig. 4.**
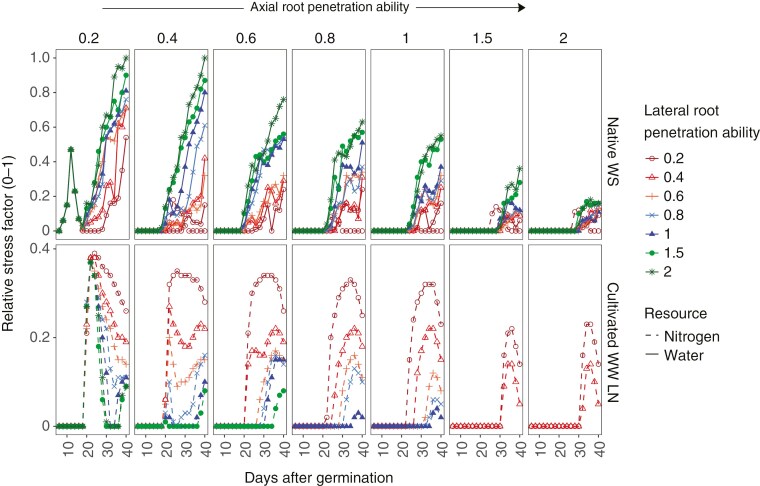
Maize root phenotypes with greater lateral root penetration ability experienced greater water stress in native undisturbed soil with dry topsoil and less N stress in irrigated cultivated soil with low N. Water and N stress as it develops over time in sandy loam Inceptisols under two management scenarios, namely a native undisturbed soil with dry topsoil (Native WS) and an irrigated cultivated soil with low N (Cultivated WW LN) in a low CO_2_ environment (270 ppm). Stress is calculated as 1 − (*u* − *m*)/(*o* − *m*), where *u* is resource uptake (water or nitrate), *o* is the optimal content in the plant and *m* is the minimal content in the plant; 0 indicates no stress, 1 indicates severe stress. The maize root phenotypes vary in axial and lateral root penetration ability. The panels represent data corresponding to increasing axial root penetration ability from left to right. The reduction in root elongation corresponding to each level of penetration ability is determined by the curve shown in Fig. 1A. Within each panel, phenotypes have the same axial root penetration but vary in lateral root penetration ability. The phenotypes varying in lateral root penetration ability are colour coded red to green with red having least lateral root penetration ability (most plastic phenotype) and green having greatest lateral penetration ability (least plastic). Phenotypes which did not experience any stress have been excluded from the figure.

Maize root phenotypes varied in their response to water and N limitation in both soil textures. Phenotypes in WW conditions were confirmed to have no water stress, while under WS conditions, water stress experienced by the plant reduced with increased axial root penetration ability (Fig. 4; Supplementary Data Fig. S2). Among phenotypes with the same axial root penetration ability, phenotypes with reduced lateral root penetration ability were less water stressed than phenotypes with greater lateral root penetration ability. Phenotypes in native soils had negligible N stress which reduced over time while those in cultivated irrigated soils had persistent sustained N stress which did not reduce over time, confirming that the simulation conditions are representative of native soil and cultivated irrigated soil. Among phenotypes with the same axial root penetration ability, phenotypes with reduced lateral root penetration ability were more N stressed than phenotypes with greater lateral root penetration ability (Fig. 4; Fig. S2).

### Reduced lateral root penetration ability results in more roots in deeper soil under water stress

**Fig. 5.**
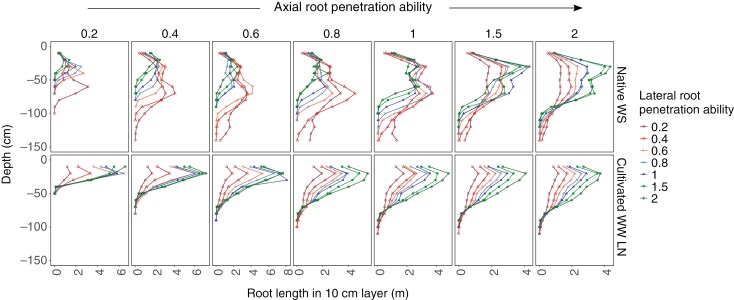
Maize root phenotypes with greater lateral root penetration ability have reduced rooting depth than phenotypes with less lateral root penetration ability. Root length distribution for phenotypes varying in axial and lateral root penetration ability at 40 d after germination in a sandy loam soil Inceptisol as a native undisturbed soil with dry topsoil (Native WS) and an irrigated cultivated soil with low N (Cultivated WW LN) in a low CO_2_ environment (270 ppm). The panels represent data corresponding to increasing axial root penetration ability from left to right. The reduction in root elongation corresponding to each level of penetration ability is determined by the curve shown in Fig. 1A. Within each panel, phenotypes have the same axial root penetration but vary in lateral root penetration ability. The phenotypes varying in lateral root penetration ability are colour coded red to green with red having least lateral root penetration ability (most plastic phenotype) and green having greatest lateral penetration ability (least plastic).

**Fig. 6.**
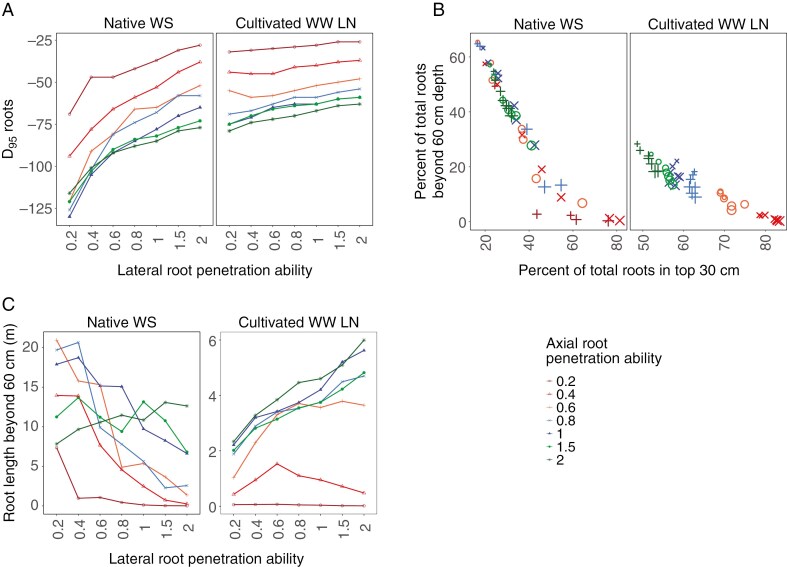
Maize root phenotypes with reduced lateral root penetration ability have more roots in deeper soil. *D*_95_, soil depth above which 95 % of root length is located, for phenotypes varying in axial and lateral root penetration ability at 40 d after germination in a sandy loam Inceptisol as a native undisturbed soil with dry topsoil (Native WS) and an irrigated cultivated soil with low N (Cultivated WW LN) in a low CO_2_ environment (A). Phenotypes with greater lateral root penetration ability have reduced *D*_95_ than phenotypes with reduced lateral root penetration ability (A). Percentage of total root length in top 30 cm and below 60 cm in a loam and sandy loam Inceptisol as a native undisturbed soil with dry topsoil (Native WS) and an irrigated cultivated soil with low N (Cultivated WW LN). Phenotypes vary in axial and lateral root penetration ability. The size of the symbols representing different data points is proportional to lateral root penetration ability. Phenotypes varying in axial root penetration ability are colour coded red to green with red having least lateral root penetration ability (most plastic phenotype) and green having greatest lateral penetration ability (least plastic) (B). The reduction in root elongation corresponding to each level of penetration ability is determined by the curve shown in Fig. 1A. Phenotypes with less lateral root penetration ability have greater root length in deep soil (beyond 60 cm depth) than phenotypes with greater lateral root penetration ability in a native undisturbed soil with dry topsoil (Native WS) (C).

Maize root phenotypes with greater axial root penetration ability had greater rooting depth than those with reduced axial root penetration ability in both native and cultivated soils (Fig. 5; Supplementary Data Fig. S3). Among phenotypes with the same axial root penetration ability, phenotypes with greater lateral root penetration ability had greater root length in the topsoil than corresponding phenotypes with reduced lateral root penetration ability in well-watered conditions. Under water deficit, phenotypes with greater lateral root penetration had shallower rooting depth than those with less lateral root penetration ability. Rooting depth decreased with increased lateral root penetration ability among phenotypes with the same axial root penetration ability (Fig. 5; Fig. S2). In both WW and WS conditions, phenotypes with greater axial root penetration ability had greater *D*_95_, the soil depth above which 95 % of root length is located. Under water deficit, among phenotypes with the same axial root penetration ability, phenotypes with less lateral root penetration ability had greater *D*_95_ than those with greater lateral root penetration ability. There was a 1.5- to 3.75-fold increase in *D*_95_ corresponding to reduced lateral root penetration ability in native soil under water deficit (Fig. 6A; Fig. S3A). The benefit of reduced lateral root penetration under water deficit was a 1.4- to 2.5-fold increase in *D*_95_ in native soil and a 1.4- to 2.9-fold increase in *D*_95_ in cultivated sandy loam soil. The benefit of reduced lateral root penetration ability under water deficit was more pronounced in cultivated loam soil (up to 5.2-fold increase) than in native loam soil (1.8- to 3.8-fold increase in *D*_95_). In well-watered conditions, increased axial root penetration increased *D*_95_, but there was relatively little difference in *D*_95_ with varying lateral root penetration ability, <1.6-fold in loam and sandy loam soil (Fig. 6A; Fig. S4A).

### Lateral root penetration ability determines tradeoffs between deep and shallow soil exploration

There was a tradeoff between root foraging in shallow vs. deep soil domains. Maize root phenotypes with reduced lateral root penetration ability had a greater proportion of roots in deeper soil than in shallow soil (Fig. 6B; Supplementary Data Fig. S4B). The tradeoff was seen clearly under water deficit in native sandy loam soil, but was not as evident as in the native loam soil. We looked at root distribution in the top 30 cm vs. that beyond 60 cm. Phenotypes in sandy loam soil had much deeper roots than those in loam soil and so the differences were not as evident at the depths shown. However, under WS conditions in cultivated soils, root impedance imposed by the greater strength of the loam soil resulted in very clear tradeoff in root distribution (Fig. S4B). In well-watered conditions, relative root distribution in topsoil and deeper soil was determined only by axial root penetration ability; variation in lateral root penetration ability made hardly any difference.

Maize root phenotypes with reduced lateral root penetration ability had greater root length in deeper soil in native soil with drying topsoil than phenotypes with greater lateral root penetration ability (Fig. 6C; Supplementary Data Fig. S4C). Root length in deeper soil reduced with increased lateral root penetration ability.

### Plasticity of root growth in response to mechanical impedance affects root foraging

**Fig. 7.**
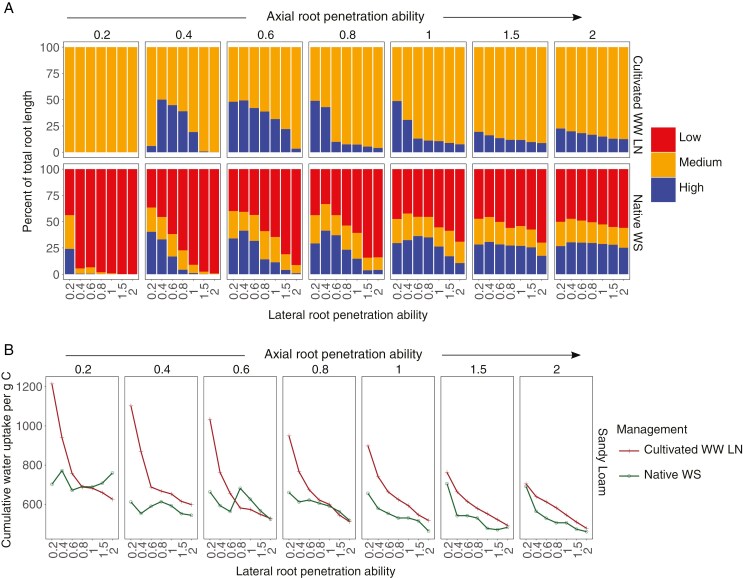
Percentage of total root length distributed in layers of soil grouped by water availability at the root surface in a sandy loam (A) Inceptisol as a native undisturbed soil with dry topsoil (Native WS) and an irrigated cultivated soil with low N (Cultivated WW LN) in a low CO_2_ environment (270 ppm). Phenotypes vary in axial and lateral root penetration ability. The reduction in root elongation corresponding to each level of penetration ability is determined by the curve shown in Fig. 1A. The panels represent data corresponding to increasing axial root penetration ability from left to right. Cumulative water uptake per gram carbon invested in roots over 20 d by maize root phenotypes varying in axial and lateral root penetration ability in a sandy loam Inceptisol as a native undisturbed soil with dry topsoil (Native WS) and an irrigated cultivated soil with low N (Cultivated WW LN) in a low CO_2_ environment (270 ppm) (B).

Under water deficit, phenotypes with greater lateral root penetration ability allocated more root length in regions with low water availability (Fig. 7A; Supplementary Data Fig. S5A). Phenotypes with less lateral root penetration ability had less than 50 % of roots in regions with low water availability. With increased lateral root penetration, more than 75 % of the roots were in regions with low water availability with a corresponding reduction in the proportion of roots in regions with greater water availability. Phenotypes with greater lateral root penetration are less efficient in water uptake and have reduced water uptake for the same amount of carbon invested in roots (Fig. 7B; Fig. S5B). By restricting root growth into strong soil domains, plants are able to reallocate resources to root growth into soil domains where the balance between carbon expenditure and water acquisition is improved.

### The fitness landscape for root adaptations to impedance has changed during crop domestication

**Fig. 8.**
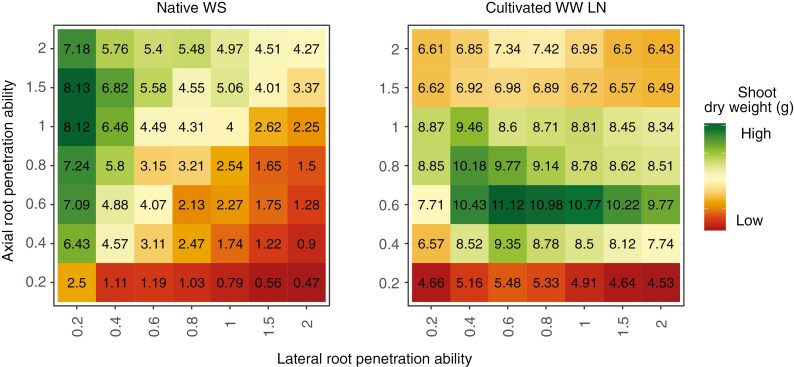
Shoot biomass at 40 d after germination in maize phenotypes varying in axial and lateral root penetration ability in a sandy loam Inceptisol as a native undisturbed soil with dry topsoil (Native WS) and an irrigated cultivated soil with low N (Cultivated WW LN) in a low CO_2_ environment (270 ppm).

Phenotypes with greater axial root penetration ability had greater shoot dry weight in water deficit (Fig. 8; Supplementary Data Fig. S6). Among phenotypes with similar axial root penetration ability, phenotypes with reduced lateral root penetration ability had greater shoot dry weight than phenotypes with greater lateral root penetration ability in water deficit in both native and cultivated soils. In soils with no water limitation, maximum shoot dry weight was attained by phenotypes with axial root penetration ability that was much less than that needed in water deficit. Increased axial root penetration beyond the optimal penetration ability resulted in reduction in shoot biomass. Cultivated irrigated soils were simulated to include two conditions of N availability: (1) cultivated well-watered soil with low N, representative of a native soil converted to cultivated agriculture which has lost organic matter and associated nutrients, as occurred in Neolithic agriculture; and (2) cultivated well-watered soils with greater N resulting from addition of fertility inputs. The optimum shifted towards greater lateral root penetration ability when N availability was suboptimal and water availability was optimal. This shift towards greater lateral root penetration ability in irrigated soils with low N was more pronounced in the sandy loam soil than in the loam soil. With increased N availability, the optimal phenotype moved slightly towards less lateral root penetration ability than needed under low N.

### Utility of the stop signal in future climates

We included a scenario with elevated atmospheric CO_2_ to assess the utility of the stop signal in future climates. A CO_2_ concentration of 270 ppm in the atmosphere representative of CO_2_ levels present during domestication (Ahn *et al.*, 2012) was used in the default simulations, and this was increased to 500 ppm to represent the projected concentration in 2050 (Prentice *et al.*, 2001). All other parameters were held constant. While the patterns in shoot productivity in response to varying axial and lateral root penetration ability were the same as those under the default CO_2_ concentration, phenotypes had greater shoot biomass under greater CO_2_ than corresponding phenotypes in default CO_2_ (Supplementary Data Figs S6 and S7). The only exception to this pattern was seen in cultivated irrigated sandy loam soil with high N, where shoot dry weight was reduced (Figs S6 and S7). The patterns in shoot productivity were similar even under CO_2_ levels representative of current levels of CO_2_ (402.9 ppm) (Figs S8 and S9).

## DISCUSSION

This study was conducted to elucidate the adaptive utility of the root ‘stop signal’, i.e. attenuation of root growth in response to high soil strength. We hypothesized that the stop signal is advantageous in native soils, in which water deficit causes drying and hardening of the topsoil, by diverting root foraging to softer, wetter subsoil. We also hypothesized that the fitness landscape of the stop signal changed in Neolithic agriculture during crop domestication with the advent of soil cultivation and irrigation, which resulted in less water stress but greater N stress. Our results support these hypotheses, providing *in silico* evidence for the utility of the stop signal when water is limited, as is common in native soil environments and is increasingly common in rainfed agriculture, but its utility is reduced when water limitation is removed, as occurred with the advent of irrigation in Neolithic agriculture.

We used *OpenSimRoot* to assess the utility of the stop signal in contrasting environments. *OpenSimRoot* is a heuristic functional–structural plant model which is useful in understanding several aspects of root/soil interactions (Ajmera *et al.*, 2022; Strock *et al.*, 2022; Schäfer *et al.*, 2022; Lopez-Valdivia *et al.*, 2023) and root phenotypes (Nielsen *et al.*, 1997; Rangarajan and Lynch, 2021). Heuristic models are useful primarily for the insights they provide rather than for their ability to predict empirical outcomes (Wullschleger *et al.*, 1994; Niklas, 2008; Yin and Struik, 2008; Zhu *et al.*, 2016), placing emphasis on the validity of underlying processes and parameters rather than agreement with empirical measurements (Dunbabin *et al.*, 2013). Several root models exist which couple soil biophysical processes with root growth and consequently plant growth responses (Wu *et al.*, 2007; Moraes *et al.*, 2018; Strock *et al.*, 2022). The strength of *OpenSimRoot* lies in its ability to simulate the interaction of root growth and soil processes dynamically in time and space while considering the effects of plant resource availability and allocation and its link with water and nutrient capture, making it apt for our study. Our results support the hypothesis that the stop signal has adaptive utility under drought stress. Phenotypes sensitive to greater soil impedance resulting in reduced root elongation in dry topsoils can allocate resources to root growth in deeper soil with greater water availability. In Neolithic soil environments in which crop domestication occurred, greater water availability from irrigation as well as reduced N availability from soil degradation transformed the selection environment for root phenotypes, resulting in diminished value for a stop signal, since lateral root elongation is important for N capture.

### Plasticity in response to mechanical impedance results in tradeoffs between deep and shallow soil exploration in native soils

Root phenotypes with more axial root penetration were beneficial in water stress conditions where the topsoil was dry and water was available in deeper soil domains, while root phenotypes with increased lateral root penetration ability were detrimental (Fig. 8). Root systems with strong penetration ability in soils with high soil strength are generally considered to be advantageous for soil exploration and resource acquisition (Cairns *et al.*, 2004; Gao *et al.*, 2016; Colombi *et al.*, 2018; Chen *et al.*, 2022). Plant fitness, however, is determined by resource allocation to many root axes and root classes exploiting different soil domains. While axial root penetration ability regulates maximum rooting depth, lateral root penetration ability regulates the distribution of root length in the soil profile as well as the maximum rooting depth (Fig. 5). Lateral roots form the bulk of the root system and are the main contributor to soil resource capture. Lateral roots are highly plastic and change the shape of the root system in response to environmental conditions. Although water deficit can repress lateral root growth (Dowd *et al.*, 2019), soil drying can induce lateral root elongation (Ito *et al.*, 2006), suggesting variation in plasticity of lateral root growth in response to water availability. Architectural and anatomical phenotypes vary among root classes and nodal positions in maize (York *et al.*, 2015; Yang *et al.*, 2019; Schneider *et al.*, 2021). Maize genotypes vary for root response to ethylene-induced root thickening, a well-known response to mechanical impedance (Vanhees *et al.*, 2022). MCS, a phene associated with enhanced root penetration ability, was found in maize axial roots but not in lateral roots and in older nodal roots (Schneider *et al.*, 2021) confirming differences in penetration ability among root classes.

Variation in plasticity in response to soil impedance among root classes results in a strong tradeoff between deep and shallow exploration (Fig. 6B). Increased lateral root penetration ability results in greater root length in the hard, dry topsoil (Fig. 6B). Investing in production of roots in regions with low resource availability incurs high carbon costs with little benefit. Root phenotypes that respond to dry soils with reduced root elongation in hard soils due to low penetration ability can allocate resources to deeper rooting, unlike phenotypes that have unhindered root growth in hard dry soil. Tradeoffs between shallow and deep soil exploration affect plant productivity by determining the capture of multiple resources with varying spatiotemporal availability (Lynch, 2013, 2022*a*, 2022*b*). Root architectural phenotypes that increase topsoil exploration improve P acquisition but incur tradeoffs for subsoil resources such as N and water (Lynch, 2018, 2022b). Tradeoffs in the capture of topsoil and subsoil resources due to varying number of axial roots, lateral root branching density and root growth angle are evident (Ho *et al.*, 2004; Postma *et al.*, 2014; Dathe *et al.*, 2016; Rangarajan *et al.*, 2018, 2022). In this study we provide evidence that tradeoffs in shallow and deep exploration can result from differences in penetration ability of different root classes, as shown previously for N capture (Strock *et al.*, 2022).

Deep soil exploration is important under drought (Manschadi *et al.*, 2006; Lopes and Reynolds, 2010; Wasson *et al.*, 2012; Lynch, 2018, 2022b). Several root phenotypes promote deeper rooting including reduced number of axial and lateral roots, steep growth angles and anatomical phenotypes that reduce the metabolic cost of soil exploration including reduced production of cortical cell files, greater loss of cortical parenchyma through formation of root cortical aerenchyma and root cortical senescence, and larger cortical cell size (Lynch, 2013, 2018; Lynch and Wojciechowski, 2015; Lynch *et al.*, 2021). Our study shows that while greater axial root penetration ability is a phenotype that enables deeper rooting, actual rooting depth is largely influenced by lateral root penetration ability through competition for internal resources among root axes.

### Value of root plasticity declined during domestication

A shallow root system is advantageous when conditions are favourable and resources are readily available in the topsoil (Lynch, 2013, 2022b). Shallow roots that favour topsoil foraging due to reduced soil strength near the soil surface can exploit water availability close to the surface from precipitation, and greater nutrient availability in the topsoil (Schenk, 2006; Lynch 2011, Lynch, 2019, 2022b). The advent of irrigation increased water availability in the topsoil. Cultivation and irrigation resulted in reduced N availability, due to topsoil erosion, microbial oxidation of organic matter from tillage, as well as leaching of available N, thereby imposing a different set of selection pressures on root phenotypes. Nitrogen is a mobile soil resource that varies spatiotemporally and therefore colocalization of roots and N availability determines N capture. Under N limitation root phenotypes with greater lateral root penetration ability (reduced plasticity) and therefore longer lateral roots are advantageous (Fig. 8). Phenotypes with the ability to allocate more root foraging to the topsoil through increased lateral root penetration ability have a competitive advantage over deep-rooted phenotypes under favourable soil conditions due to less soil strength near the soil surface, water availability close to the surface from precipitation and greater nutrient availability in the topsoil. Since plasticity is adaptive in variable or unpredictable environments (Schneider and Lynch, 2020), human stabilization of the soil–water environment through irrigation relaxed the selective pressure that maintained plasticity to mechanical impedance. Roots have been under indirect selection by direct improvement of yield-related above-ground phenotypes in target environments while genetic diversity for root phenotypes in modern crop gene pools was reduced by domestication (Lynch, 2018; Voss-Fels *et al.*, 2018). Our study provides *in silico* evidence that there is loss in the utility of plasticity to mechanical impedance in cultivated soils under irrigation.

Reduced plasticity is not beneficial under high-input systems (Supplementary Data Fig. S7). Having too many roots is costly; parsimony with reduced plasticity in response to resource availability may have utility in high-input systems (Lynch, 2018). Shallow distribution of root length in the soil profile, as may be caused by reduced plasticity, results in an increased drought hazard (Manschadi *et al.*, 2006; Colombi *et al.*, 2018; Lynch, 2018). It is possible that in natural systems, neighbouring plants with deeper and shallower roots may benefit each other through root-mediated hydraulic redistribution, so shallow and deep phenotypes coexisted and thrived (Brooker *et al.*, 2015). Fitness benefits resulting from variation in architectural phenotypes among taxa may have been tapped effectively during domestication by polyculture (Zizumbo-Villarrael and Colunga-GarciaMarin, 2010) in which selection for complementary growth habits and soil foraging strategies would have been favoured (Rubio *et al.*, 2001; Postma and Lynch, 2012; Zhang *et al.*, 2014; Burridge *et al.*, 2020). Change in maize cultivation from low fertilizer inputs and low population densities to intensive fertilization and dense populations has resulted in indirect selection of maize root systems adapted to intense competition for N (York *et al.*, 2015).

### Role of plasticity in future soils and climate

Adoption of sustainable agriculture practices such as conservation agriculture along with climate-induced change in water availability results in features similar to native soils (Lynch *et al.*, 2022). More plastic root phenotypes that avoid hard, dry soil domains and efficiently explore deeper soil domains with greater water are advantageous (Supplementary Data Fig. S7). Increased plasticity conferred by the stop signal is advantageous even in future conditions with increased atmospheric CO_2_ (Fig. S8). Domestication and associated changes in the soil environment have been pivotal in shaping the evolution of root architecture. Soil continues to change with climate change and agricultural practices and so root systems of future crops should be selected for adaptations to contrasting soil environments in different agroecosystems (Lynch *et al.*, 2022).

### Implications for crop breeding

Our results have implications for crop breeding for current and future agroecosystems. The data presented in this paper were generated with an atmospheric CO_2_ concentration characteristic of the Neolithic, i.e. 270 ppm, corresponding to the advent of cultivated agriculture and crop domestication. In the Supplementary Data files we present an equivalent set of results but at current atmospheric CO_2_ concentration, i.e. 402.9 ppm, in order to assess the importance of these processes for modern agriculture. Results at the current CO_2_ concentration are comparable to those at the Neolithic CO_2_ concentration, indicating that the role of the stop signal for plant fitness under drought and N stress is relevant today. As noted above, genotypic variation for root responses to impedance have been reported in multiple crop species, including maize (Vanhees *et al.*, 2022). In general terms, global agroecosystems can be divided between high-input systems with N fertilization, and low-input systems with limited N fertilization. For field crop production, in both types of systems water deficit is a primary limitation, which is projected to intensify in coming decades as a result of global climate change, soil degradation and degradation of freshwater resources. Conservation agriculture, increasingly adopted in high-input systems, is returning soils to the physical characteristics of native soils (Lynch *et al*, 2022). Our results suggest that independent of N fertility, under water stress the stop signal of growth cessation in response to soil hardness is useful, by allowing root foraging to be allocated to softer, wetter, deeper soil domains. Modern crops and modern crop breeding have often been selected without significant water stress. There are opportunities to increase the growth plasticity of lateral roots in response to soil hardness through crop breeding. In low-fertility systems, lateral root elongation is useful for exploitation of N resources, which are spatiotemporally dynamic, when sufficient water is available. With severe water stress, the stop signal is useful in present as well as future climatic conditions.

## SUPPLEMENTARY DATA

Supplementary data are available at *Annals of Botany* online and consist of the following.

Fig. S1: High-resolution image of representative maize root phenotypes at 40 d after germination in a loam Inceptisol as a native undisturbed soil with dry topsoil (A) and an irrigated cultivated soil with low N (B). Fig. S2: Maize root phenotypes with greater lateral root penetration ability experienced greater water stress in native undisturbed soil with dry topsoil and less N stress in irrigated cultivated soil with low N. Water and N stress as it develops over time in a loam Inceptisol under two management scenarios, namely a native undisturbed soil with dry topsoil (Native WS) and an irrigated cultivated soil with low N (Cultivated WW LN) in a low CO_2_ environment (270 ppm). Fig. S3: Maize root phenotypes with greater lateral root penetration ability have reduced rooting depth than phenotypes with lower lateral root penetration ability. Fig. S4: Maize root phenotypes with reduced lateral root penetration ability have more roots in deeper soil. Fig. S5: Percentage of total root length distributed in layers of soil grouped by water availability at the root surface in a loam Inceptisol as a native undisturbed soil with dry topsoil (Native WS) and an irrigated cultivated soil with low N (Cultivated WW LN) in a low CO_2_ environment (270 ppm). Fig. S6: Shoot biomass at 40 d after germination in maize phenotypes varying in axial and lateral root penetration ability in a loam Inceptisol as a native undisturbed soil with dry topsoil (Native WS) and an irrigated cultivated soil with low N (Cultivated WW LN) in a low CO_2_ environment (270 ppm). Fig. S7: Water and nutrient stress as it develops over time in loam (A) and sandy loam (B) Inceptisol under four soil management scenarios, namely an irrigated cultivated soil with high N (Cultivated WW HN), cultivated soil with dry topsoil and high N (Cultivated WS HN), well-watered native soil (Native WW), and cultivated soil with dry topsoil and low N (Cultivated WS LN) in a low CO_2_ environment (270 ppm). Fig. S8: Maize root phenotypes with greater lateral root penetration ability have reduced rooting depth than phenotypes with lower lateral root penetration ability. Fig. S9: Maize root phenotypes with reduced lateral root penetration ability have greater root length in deeper soil (beyond 60 cm depth) under water limitation. Fig. S10: Percentage of total roots distributed in layers of soil grouped by water availability at the root surface in a loam (A) and sandy loam Inceptisol (B) under four soil management scenarios as an irrigated cultivated soil with high N (Cultivated WW HN), cultivated soil with dry topsoil and high N (Cultivated WS HN), well-watered native soil (Native WW), and cultivated soil with dry topsoil and low N (Cultivated WS LN) in a low CO_2_ environment (270 ppm). Fig. S11: Shoot biomass at 40 d after germination in phenotypes varying in axial and lateral root penetration resistance in a loam (A) and sandy loam Inceptisol (B) under four soil management scenarios as an irrigated cultivated soil with high N (Cultivated WW HN), cultivated soil with dry topsoil and high N (Cultivated WS HN), well-watered native soil (Native WW), and cultivated soil with dry topsoil and low N (Cultivated WS LN) in a low CO_2_ environment. Fig. S12: Shoot biomass at 40 d after germination in phenotypes varying in axial and lateral root penetration ability in a loam (A) and sandy loam Inceptisol (B) as a native undisturbed soil with dry topsoil (Native WS), and an irrigated cultivated soil with low N (Cultivated WW LN) in a high CO_2_ environment (500 ppm).

Fig. S13: Shoot biomass at 40 d after germination in phenotypes varying in axial and lateral root penetration ability in a loam (A) and sandy loam Inceptisol (B) as an irrigated cultivated soil with high N (Cultivated WW HN), cultivated soil with dry topsoil and high N (Cultivated WS HN), well-watered native soil (Native WW), and cultivated soil with dry topsoil and low N (Cultivated WS LN) in a high CO_2_ environment (500 ppm). Fig. S14: Shoot biomass at 40 d after germination in maize phenotypes varying in axial and lateral root penetration ability in a loam and sandy loam Inceptisol as a native undisturbed soil with dry topsoil (Native WS) and an irrigated cultivated soil with low N (Cultivated WW LN) in an environment with 402.9 ppm CO_2_. Fig. S15: Shoot biomass at 40 d after germination in phenotypes varying in axial and lateral root penetration ability in a loam (A) and sandy loam Inceptisol (B) under four soil management scenarios as an irrigated cultivated soil with high N (Cultivated WW HN), cultivated soil with dry topsoil and high N (Cultivated WS HN), well-watered native soil (Native WW), and cultivated soil with dry topsoil and low N (Cultivated WS LN) in an environment with 402.9 ppm CO_2._ Table. S1: Bulk density *D*_b_ and the van Genutchen parameters θ_r_ (residual water content), θ_s_ (saturated water content), α, *n* and the saturated hydraulic conductivity *K*_s_ for the loam and sandy loam Inceptisol. The parameters α and *n* describe the shape of the soil–water retention curve.

mcae201_suppl_Supplementary_Data

mcae201_suppl_Supplementary_Table_S1

## Data Availability

The input files and source code used in this study are hosted in https://figshare.com/s/5fd66984b4fe27185582
