# Supplementary material for: Did crop domestication change the fitness landscape of root response to soil mechanical impedance? An in silico analysis
Source: Ann Bot. 2024 Nov 27;136(5-6):997–1011. doi: 10.1093/aob/mcae201 (PMC12682825; doi:10.1093/aob/mcae201)
Supplement: mcae201_suppl_Supplementary_Data [file mcae201_suppl_supplementary_data.zip › Supplementary_Figures_S2___S15_combined.pdf]

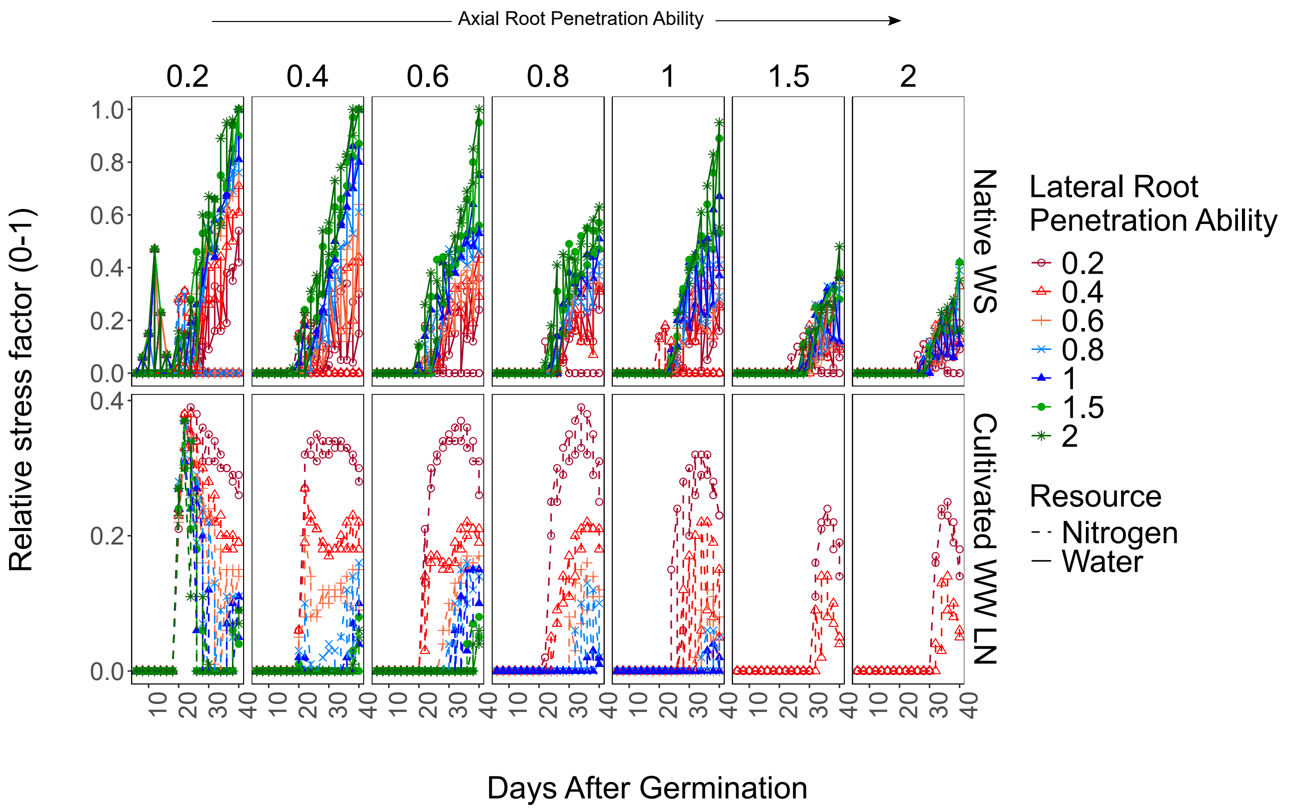

Supplementary Data Fig. S2: Maize root phenotypes with greater lateral root penetration ability experienced greater water stress in native undisturbed soil with dry topsoil and less nitrogen stress in irrigated cultivated soil with low N. Water and nitrogen stress as it develops over time in a loam Cambisol under two management scenarios namely a native undisturbed soil with dry topsoil (Native WS) and an irrigated cultivated soil with low N (Cultivated WW LN) in a low CO2 environment (270 ppm). Stress is calculated as 1-(u-m)/(o-m), where u is the resource uptake (water or nitrate), o is the optimal content in the plant and m is the minimal content in the plant. 0 indicates no stress, 1 indicates severe stress. The maize root phenotypes vary in axial and lateral root penetration ability. The panels represent data corresponding to increasing axial root penetration ability from left to right. The reduction in root elongation corresponding to each level of penetration ability is determined by the curve shown in Fig. 1(A). Within each panel, phenotypes have same axial root penetration but vary in lateral root penetration ability. The phenotypes varying in lateral root penetration ability are color coded red to green with red having least lateral root penetration ability (most plastic phenotype) to green having greatest lateral penetration ability (least plastic). Phenotypes which did not experience any stress have been excluded from the figure.

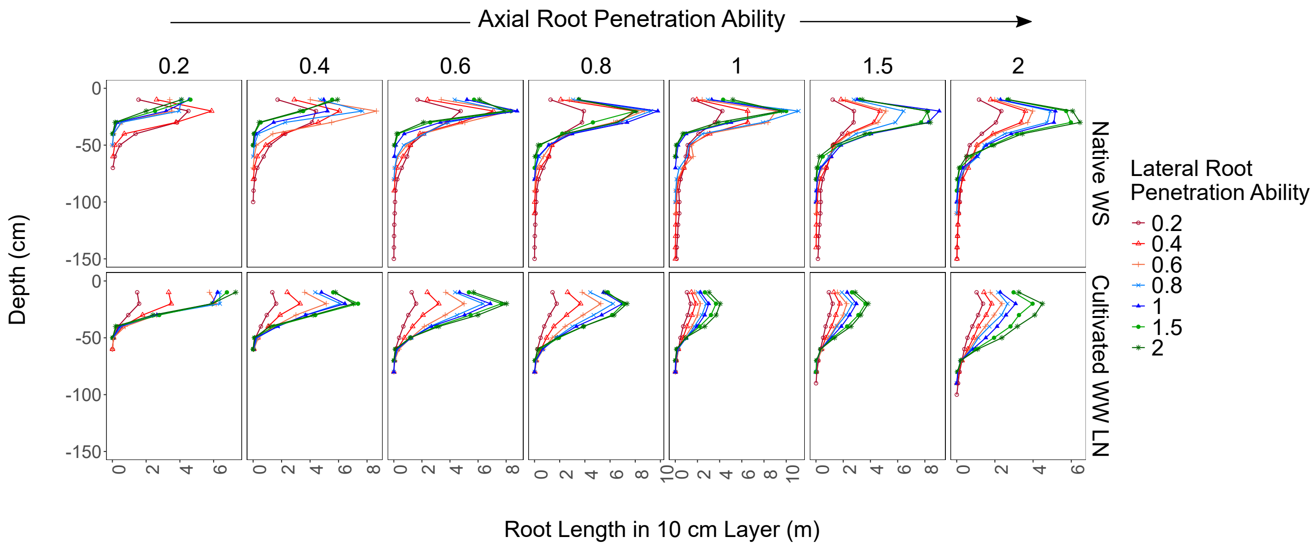

Supplementary Data Fig. S3: Maize root phenotypes with greater lateral root penetration ability have reduced rooting depth than phenotypes with lower lateral root penetration ability. Root length distribution for phenotypes varying in axial and lateral root penetration ability at 40 days after germination in a loam Cambisol as a native undisturbed soil with dry topsoil (Native WS) and an irrigated cultivated soil with low N (Cultivated WW LN) in a low CO_2 environment (270 ppm). The panels represent data corresponding to increasing axial root penetration ability from left to right. The reduction in root elongation corresponding to each level of penetration ability is determined by the curve shown in Fig. 1(A). Within each panel, phenotypes have same axial root penetration but vary in lateral root penetration ability. The phenotypes varying in lateral root penetration ability are color coded red to green with red having least lateral root penetration ability (most plastic phenotype) to green having greatest lateral penetration ability (least plastic).

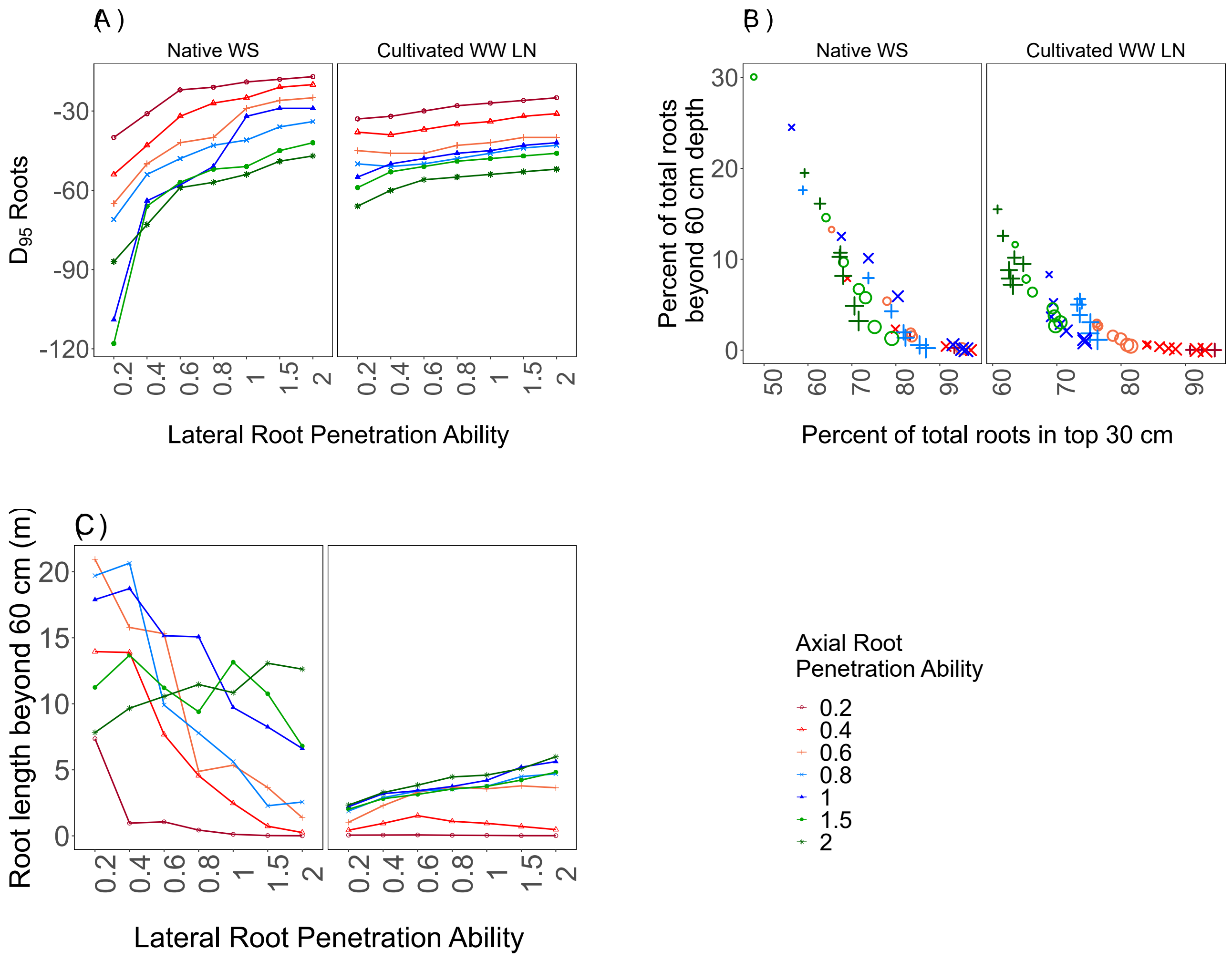

Supplementary Data Fig. S4: Maize root phenotypes with reduced lateral root penetration ability have more roots in deeper soil. D95, soil depth above which 95 % of root length is located, for phenotypes varying in axial and lateral root penetration ability at 40 days after germination in a loam Cambisol as a native undisturbed soil with dry topsoil (Native WS) and an irrigated cultivated soil with low N (Cultivated WW LN) in a low CO_2 environment (A). Phenotypes with greater lateral root penetration ability have reduced D95 than phenotypes with reduced lateral root penetration ability (A). Percent of total root length in top 30 cm and below 60 cm in a loam and sandy loam Cambisol as a native undisturbed soil with dry topsoil (Native WS) and an irrigated cultivated soil with low N (Cultivated WW LN). Phenotypes vary in axial and lateral root penetration ability. The size of the symbols representing different data points is proportional to lateral root penetration ability. Phenotypes varying in axial root penetration ability are color coded red to green with red having least lateral root penetration ability (most plastic phenotype) to green having greatest lateral penetration ability (least plastic) (B). The reduction in root elongation corresponding to each level of penetration ability is determined by the curve shown in Fig. 1(A). Phenotypes with lower lateral root penetration ability have greater root length in deep soil (beyond 60 cm depth) than phenotypes with greater lateral root penetration ability in a native undisturbed soil with dry topsoil (Native WS) (C).

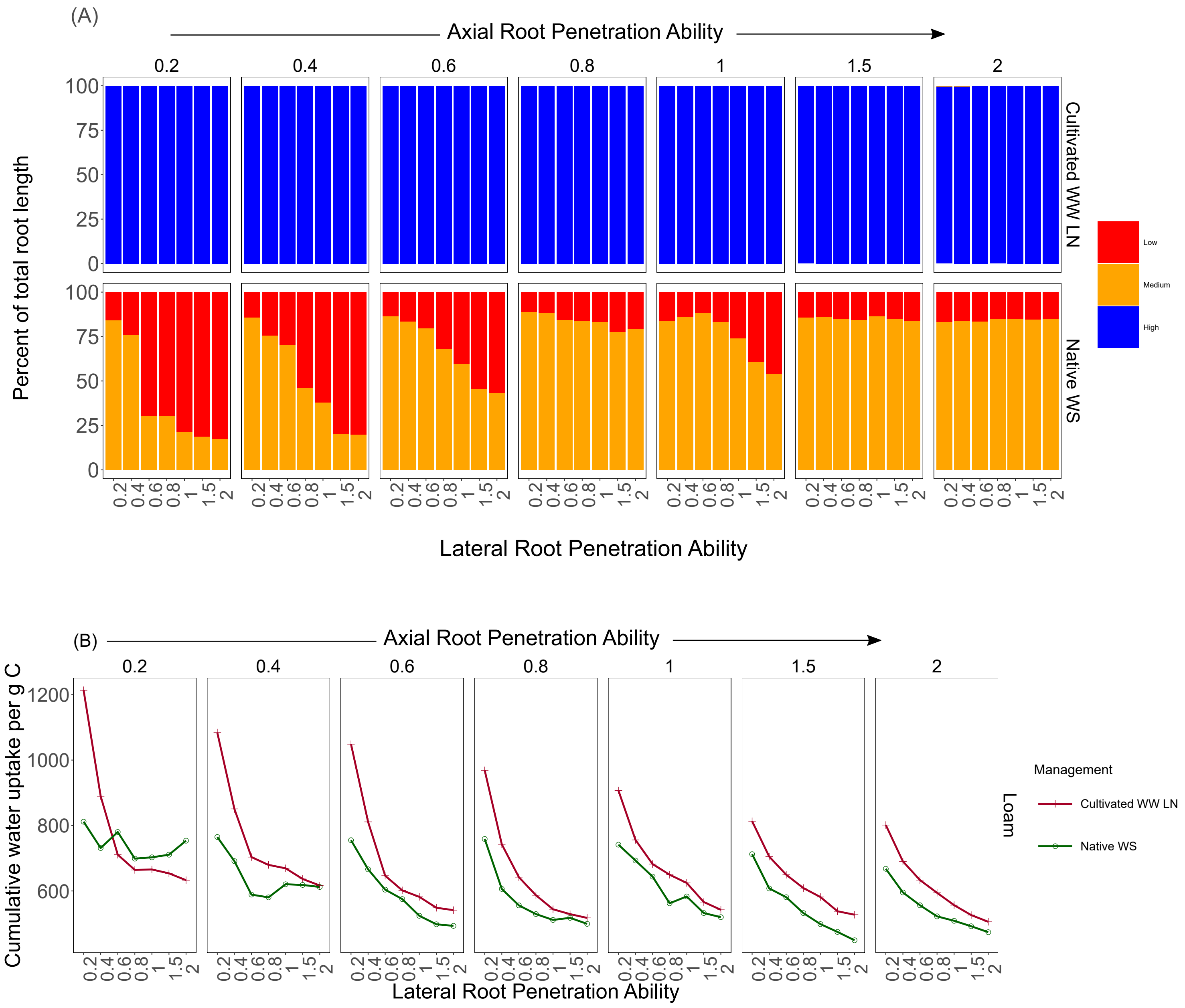

Supplementary Data FigS5: Percent of total root length distributed in layers of soil grouped by water availability at the root surface in a loam Cambisol as a native undisturbed soil with dry topsoil (Native WS) and an irrigated cultivated soil with low N (Cultivated WW LN) in a low CO_2 environment (270 ppm). (A) Phenotypes vary in axial and lateral root penetration ability. The reduction in root elongation corresponding to each level of penetration ability is determined by the curve shown in Fig. 1(A). The panels represent data corresponding to increasing axial root penetration ability from left to right. Cumulative water uptake per gram carbon invested in roots over 20 days by maize root phenotypes varying in axial and lateral root penetration ability in a loam Cambisol as a native undisturbed soil with dry topsoil (Native WS) and an irrigated cultivated soil with low N (Cultivated WW LN) in a low CO_2 environment (270 ppm) (B).

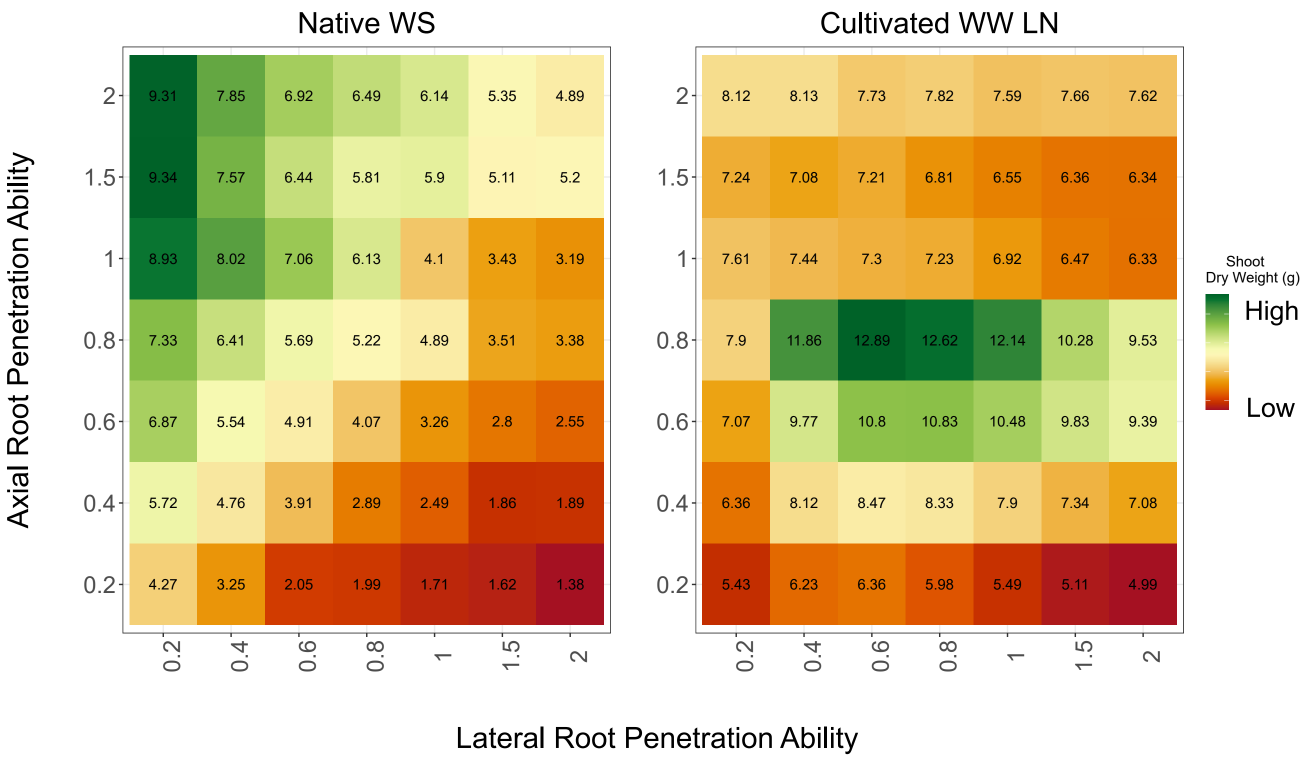

Supplementary Data Fig S6: Shoot biomass at 40 days after germination in maize phenotypes varying in axial and lateral root penetration ability in a loam Cambisol as a native undisturbed soil with dry topsoil (Native WS) and an irrigated cultivated soil with low N (Cultivated WW LN) in a low CO2 environment (270 ppm).

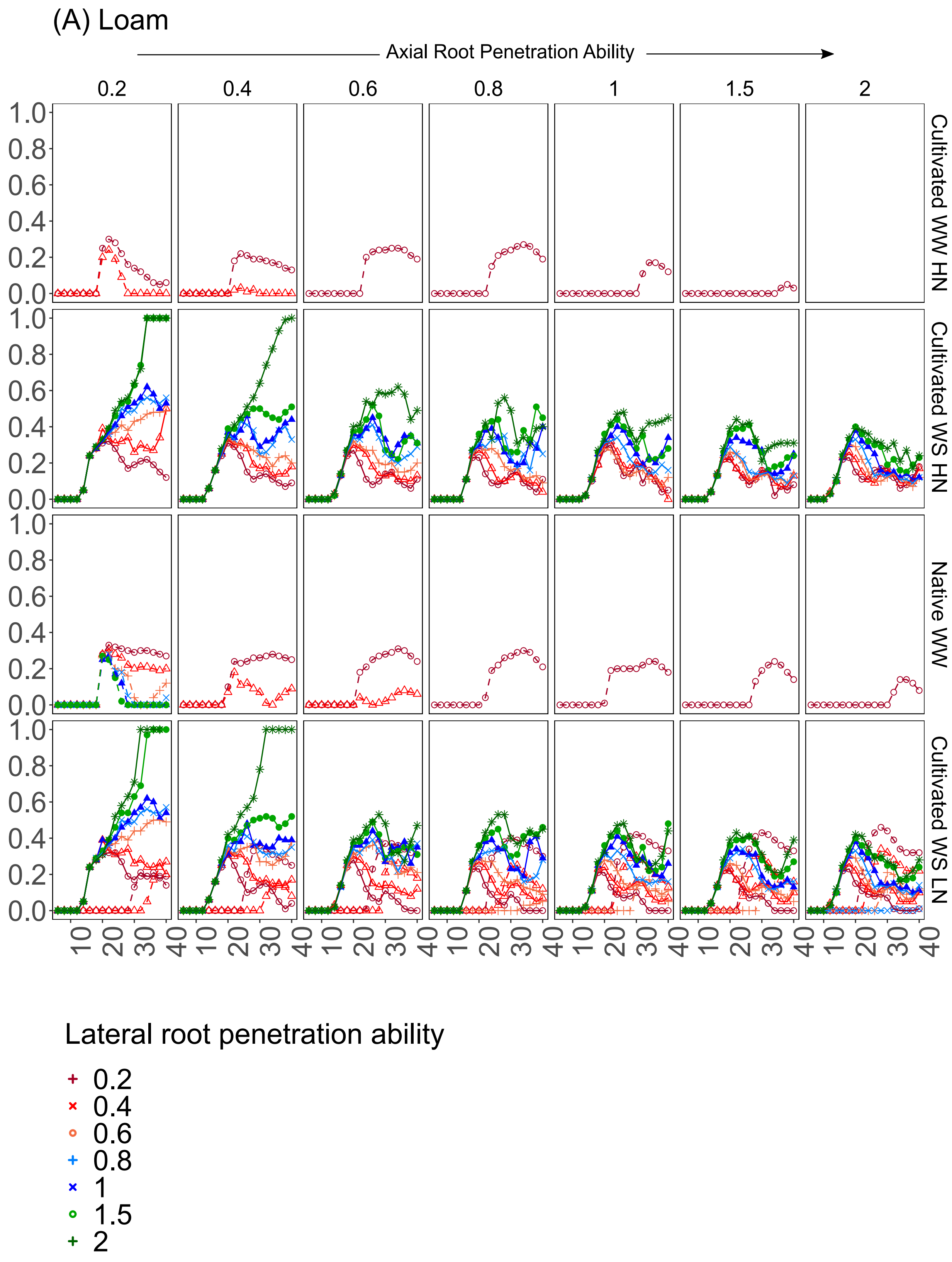

(A) Loam
Axial Root Penetration Ability
0.2
0.4
0.6
0.8
1
1.5
2
Cultivated WW HN
Cultivated WS HN
Native WW
Cultivated WS LN
1.0
0.8
0.6
0.4
0.2
0.0
10
20
30
40
Lateral root penetration ability
0.2
0.4
0.6
0.8
1
1.5
2

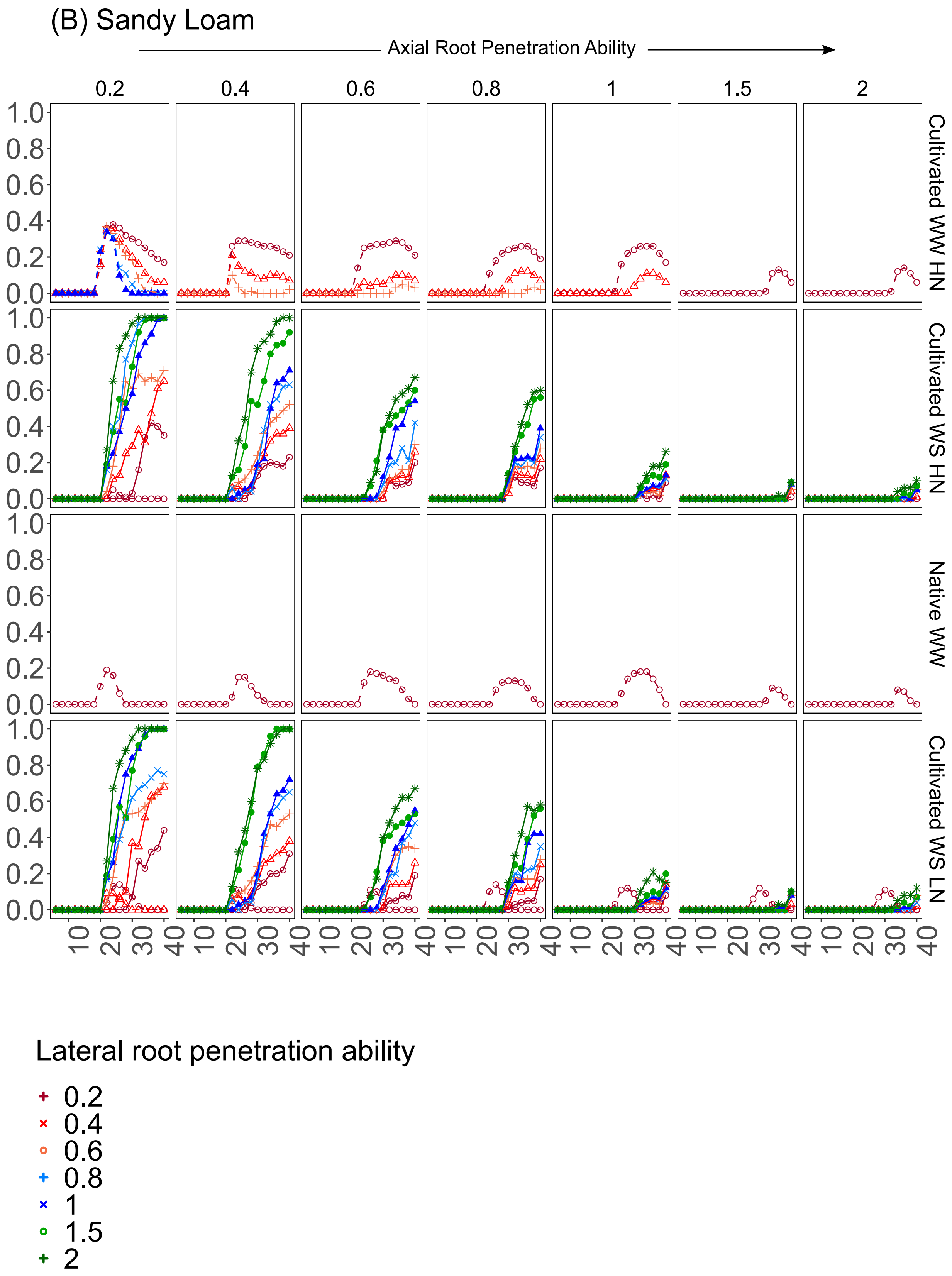
(B) Sandy Loam
Axial Root Penetration Ability
0.2
0.4
0.6
0.8
1
1.5
2
Cultivated WW HN
Cultivated WS HN
Native WW
Cultivated WS LN
1.0
0.8
0.6
0.4
0.2
0.0
10
20
30
40
Lateral root penetration ability
0.2
0.4
0.6
0.8
1
1.5
2

Supplementary Data Fig. S7: Water and Nutrient stress as it develops over time in loam (A) and sandy loam (B) Cambisol under 4 soil management scenarios namely an irrigated cultivated soil with high N (Cultivated WW HN) , cultivated soil with dry topsoil and high N (Cultivated WS HN), well-watered native soil (Native WW) , cultivated soil with dry topsoil and low N (Cultivated WS LN) in a low CO_2 environment (270 ppm). Stress is calculated as 1-(u-m)/(o-m), where u is the resource uptake (water or nitrate), o is the optimal content in the plant and m is the minimal content in the plant. 0 indicates no stress, 1 indicates severe stress. The maize root phenotypes vary in axial and lateral root penetration ability. The panels represent data corresponding to increasing axial root penetration ability from left to right. The reduction in root elongation corresponding to each level of penetration ability is determined by the curve shown in Fig. 1(A). Within each panel, phenotypes have same axial root penetration but vary in lateral root penetration ability. The phenotypes varying in lateral root penetration ability are color coded red to green with red having least lateral root penetration ability (most plastic phenotype) to green having greatest lateral penetration ability (least plastic).

(A) Loam

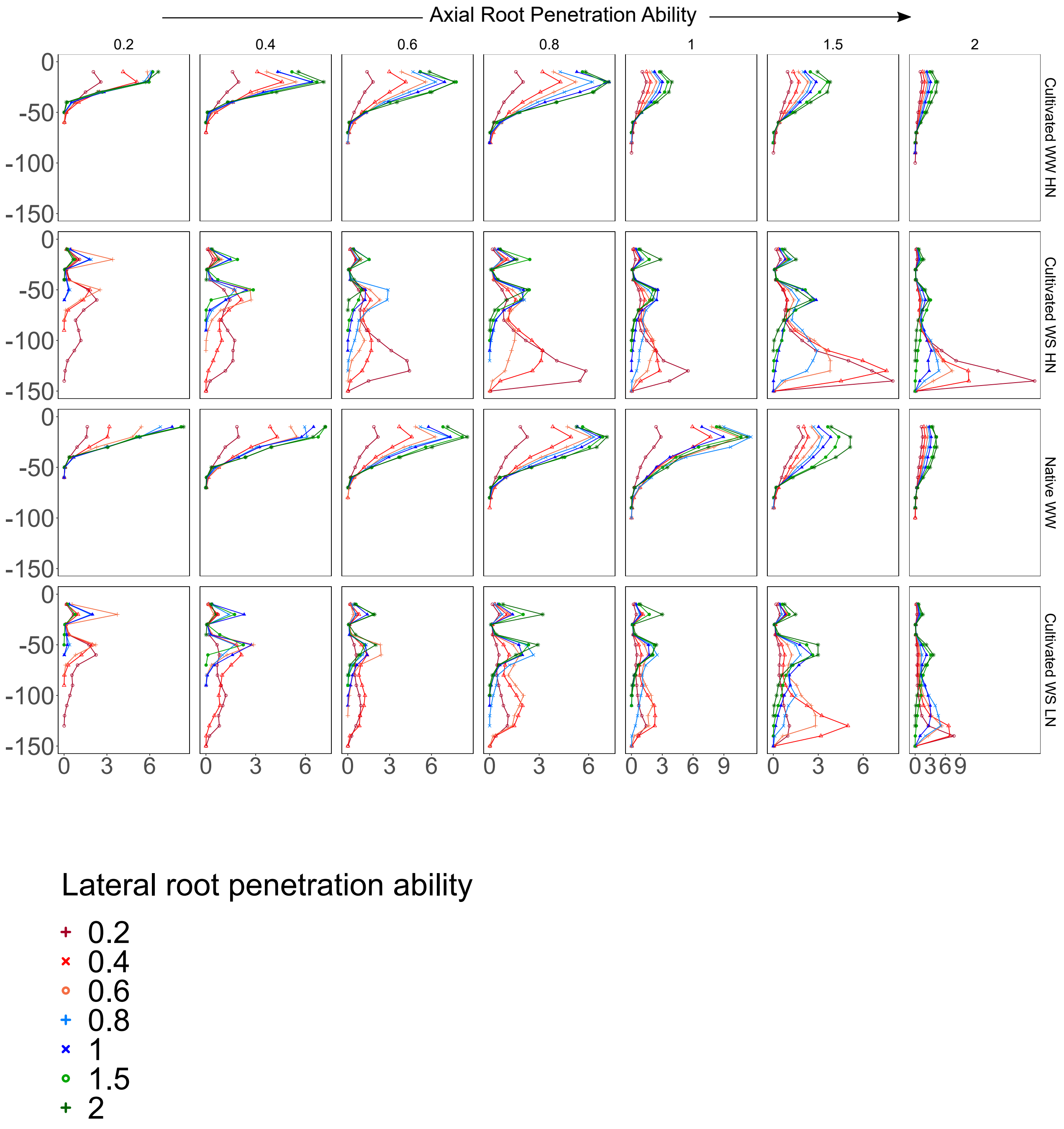

Axial Root Penetration Ability
0.2
0.4
0.6
0.8
1
1.5
2
Cultivated WW HN
Cultivated WS HN
Native WW
Cultivated WS LN
0
-50
-100
-150
0 3 6
0 3 6 9
0369
Lateral root penetration ability
0.2
0.4
0.6
0.8
1
1.5
2

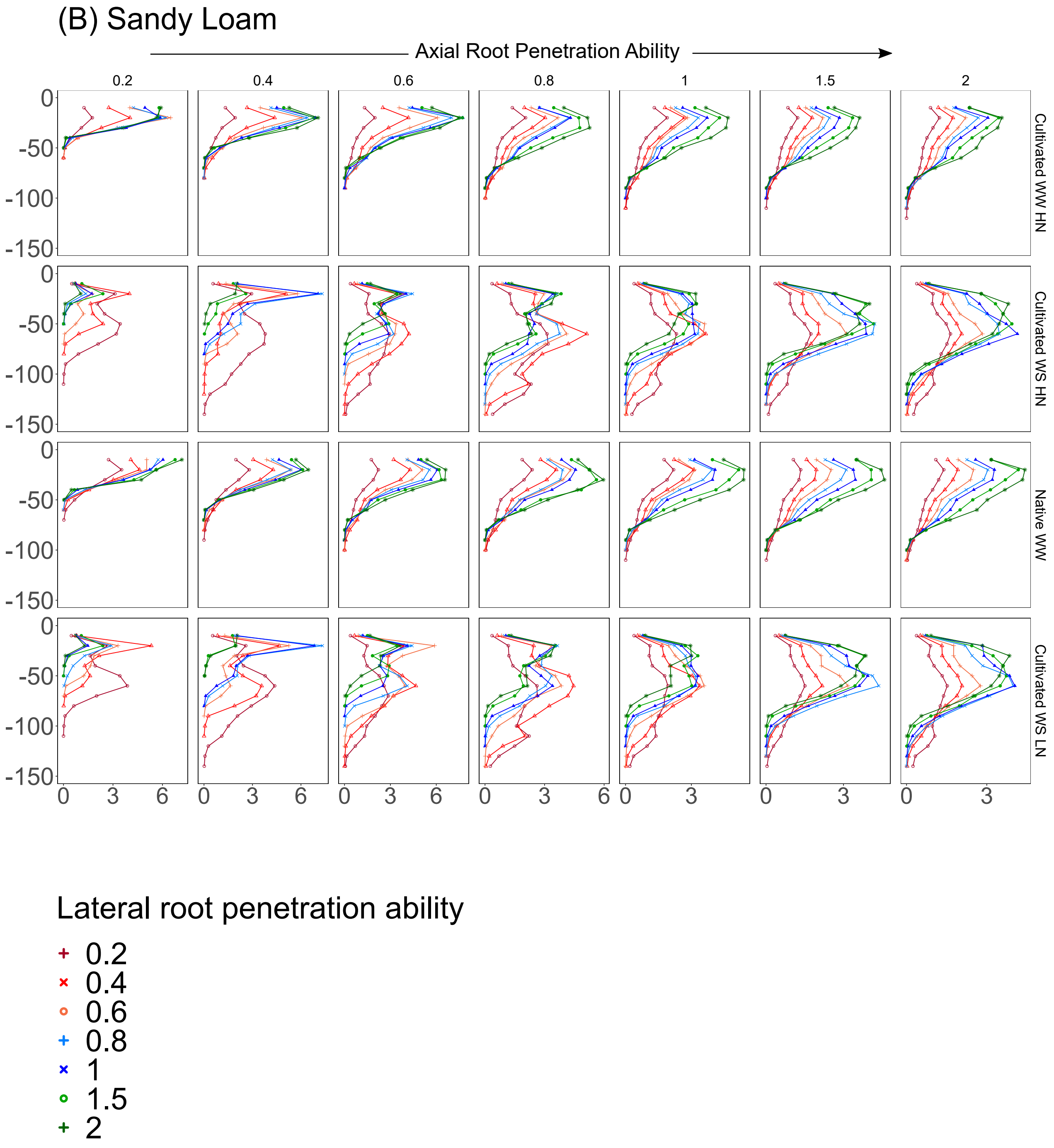
(B) Sandy Loam
Axial Root Penetration Ability
0.2
0.4
0.6
0.8
1
1.5
2
Cultivated WW HN
Cultivated WS HN
Native WW
Cultivated WS LN
0
-50
-100
-150
0
3
6
Lateral root penetration ability
0.2
0.4
0.6
0.8
1
1.5
2

Supplementary Data Fig. S8: Maize root phenotypes with greater lateral root penetration ability have reduced rooting depth then phenotypes with lower lateral root penetration ability. Root length distribution for maize root phenotypes varying in axial and lateral root penetration ability at 40 days after germination in a loam (A) and sandy loam (B) Cambisol under 4 soil management scenarios namely an irrigated cultivated soil with high N (Cultivated WW HN) , cultivated soil with dry topsoil and high N (Cultivated WS HN), well-watered native soil (Native WW) , cultivated soil with dry topsoil and low N (Cultivated WS LN) in a low CO_2 environment (270 ppm). The panels represent data corresponding to increasing axial root penetration ability from left to right. The reduction in root elongation corresponding to each level of penetration ability is determined by the curve shown in Fig. 1(A). Within each panel, phenotypes have same axial root penetration but vary in lateral root penetration ability. The phenotypes varying in lateral root penetration ability are color coded red to green with red having least lateral root penetration ability (most plastic phenotype) to green having greatest lateral penetration ability (least plastic).

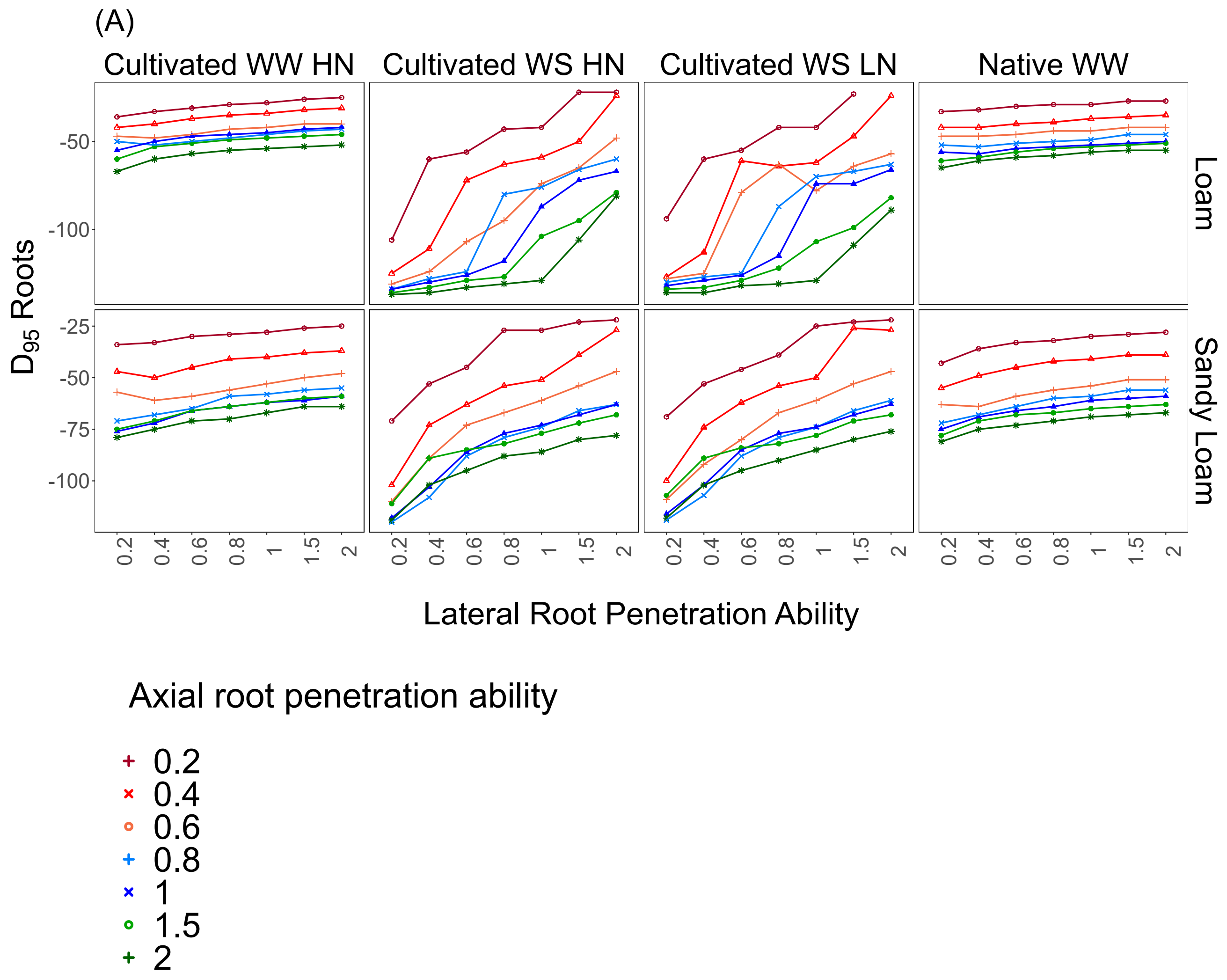
(A)
Cultivated WW HN
Cultivated WS HN
Cultivated WS LN
Native WW
Loam
Sandy Loam
D95 Roots
-25
-50
-75
-100
0.2
0.4
0.6
0.8
1
1.5
2
Lateral Root Penetration Ability
Axial root penetration ability
0.2
0.4
0.6
0.8
1
1.5
2

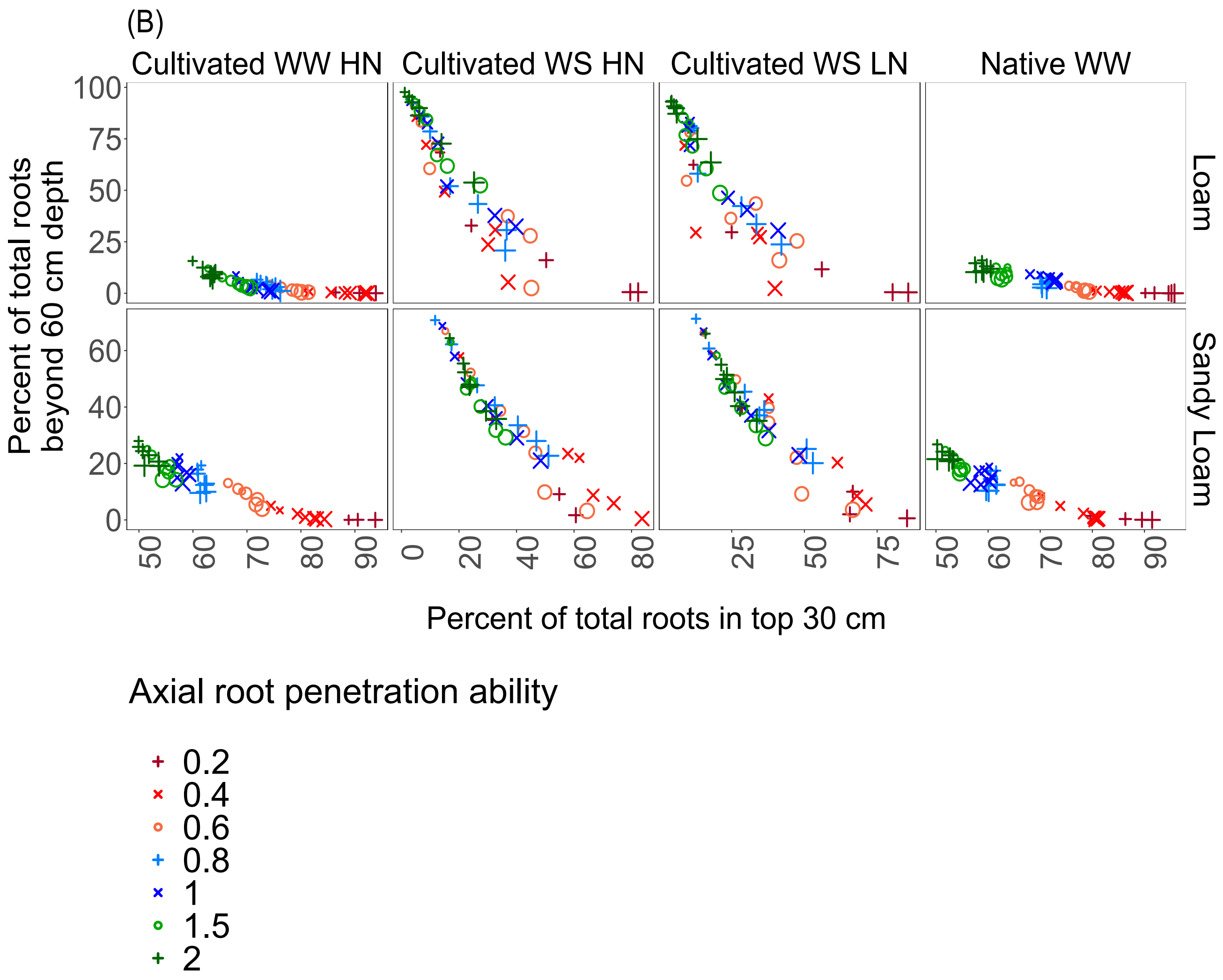

(B)
Cultivated WW HN
Cultivated WS HN
Cultivated WS LN
Native WW
Loam
Sandy Loam
Percent of total roots beyond 60 cm depth
Percent of total roots in top 30 cm
Axial root penetration ability
0.2
0.4
0.6
0.8
1
1.5
2

(C)

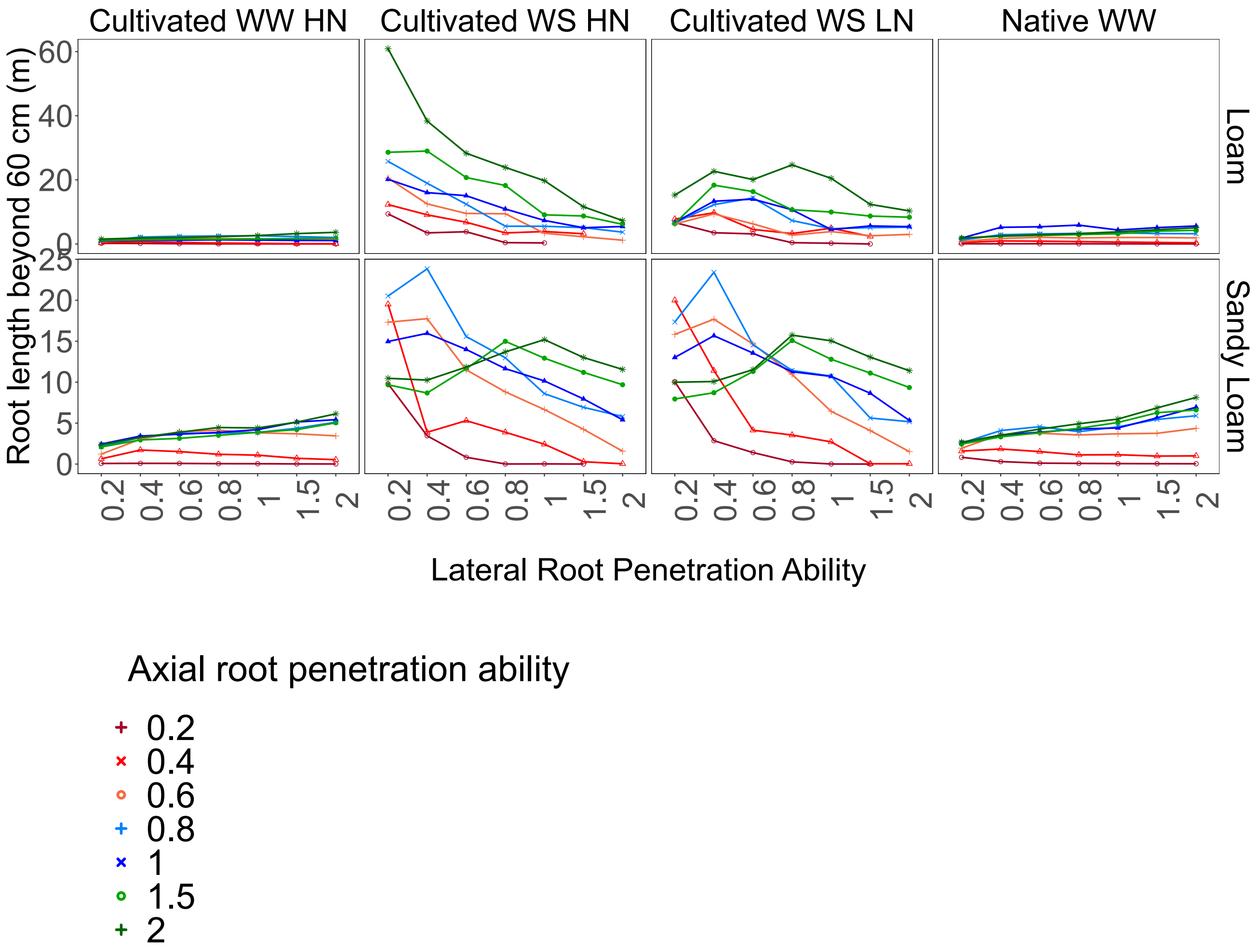

Supplementary Data Fig. S9: Maize root phenotypes with reduced lateral root penetration ability have greater root length in deeper soil (beyond 60 cm depth) under water limitation. D95, soil depth above which 95% of root length is located , for phenotypes varying in axial and lateral root penetration ability at 40 days after germination in a loam and sandy loam Cambisol under four soil management scenarios as an irrigated cultivated soil with high N (Cultivated WW HN), cultivated soil with dry topsoil and high N (Cultivated WS HN), cultivated soil with dry topsoil and low N (Cultivated WS LN), well watered native soil (Native WW) in a low CO_2 environment (270 ppm) (A). Phenotypes with greater lateral root penetration ability have reduced D95 then phenotypes with lower lateral root penetration ability (A). Percent of total root length in top 30 cm and below 60 cm in a loam and sandy loam Cambisol under four soil management scenarios as an irrigated cultivated soil with high N (Cultivated WW HN), cultivated soil with dry topsoil and high N (Cultivated WS HN), cultivated soil with dry topsoil and low N (Cultivated WS LN), well watered native soil (Native WW) in a low CO_2 environment (270 ppm) (B). Phenotypes vary in axial and lateral root penetration ability. The size of the symbols representing different data points is proportional to lateral root penetration ability. Phenotypes varying in axial root penetration ability are color coded red to green with red having least lateral root penetration ability (most plastic phenotype) to green having greatest lateral penetration ability (least plastic) (B). The reduction in root elongation corresponding to each level of penetration ability is determined by the curve shown in Fig. 1(A). Phenotypes with lower lateral root penetration ability have greater root length in deep soil (beyond 60 cm depth) than phenotypes with greater lateral root penetration ability under water stress (C).

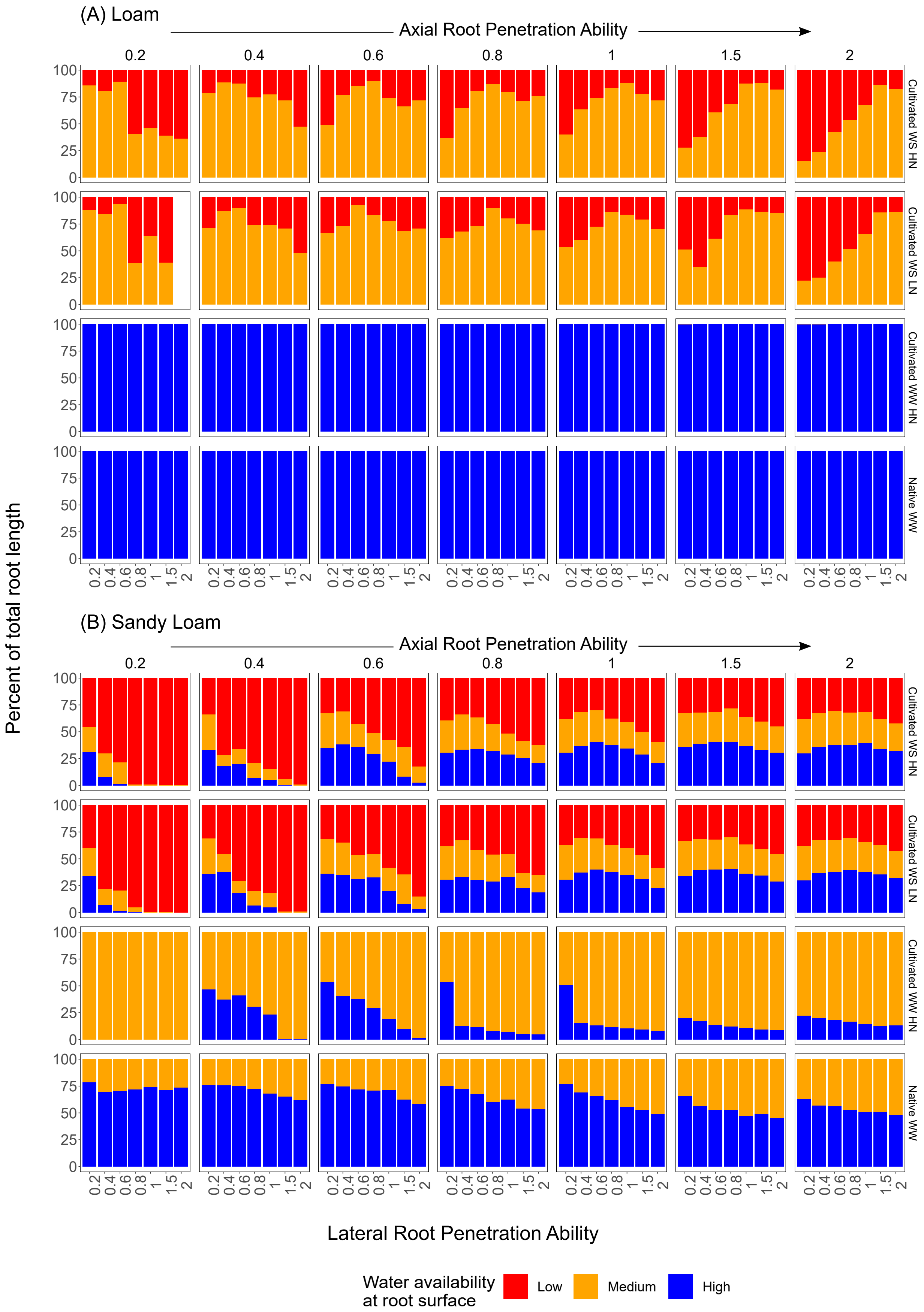

(A) Loam
Axial Root Penetration Ability
0.2
0.4
0.6
0.8
1
1.5
2
Cultivated WS HN
Cultivated WS LN
Cultivated WW HN
Native WW
(B) Sandy Loam
Percent of total root length
Lateral Root Penetration Ability
Water availability at root surface
Low
Medium
High

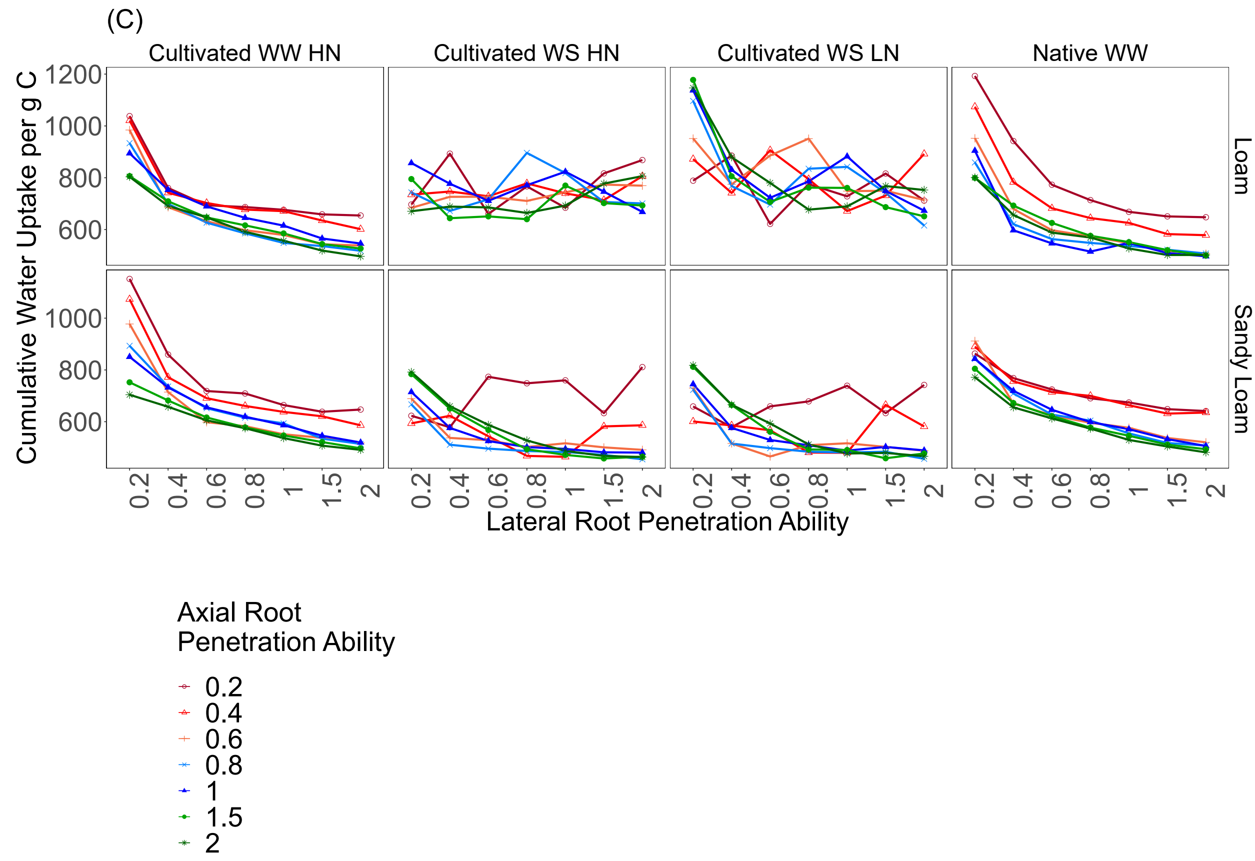

Supplementary Data Fig. S10: Percent of total roots distributed in layers of soil grouped by water availability at the root surface in a loam (A) and sandy loam Cambisol (B) under 4 soil management scenarios as an irrigated cultivated soil with high N (Cultivated WW HN) , cultivated soil with dry topsoil and high N (Cultivated WS HN), well-watered native soil (Native WW) , cultivated soil with dry topsoil and low N (Cultivated WS LN) in a low CO_2 environment (270 ppm). The maize root phenotypes vary in axial and lateral root penetration ability. The panels represent data corresponding to increasing axial root penetration ability from left to right. The reduction in root elongation corresponding to each level of penetration ability is determined by the curve shown in Fig. 1(A). Cumulative water uptake per gram carbon invested in roots over 20 days (from day 20 to 40) by maize root phenotypes varying in axial and lateral root penetration ability in a loam and a sandy loam Cambisol under 4 soil management scenarios as an irrigated cultivated soil with high N (Cultivated WW HN) , cultivated soil with dry topsoil and high N (Cultivated WS HN), well-watered native soil (Native WW) , cultivated soil with dry topsoil and low N (Cultivated WS LN) in a low CO_2 environment (270 ppm) (C).

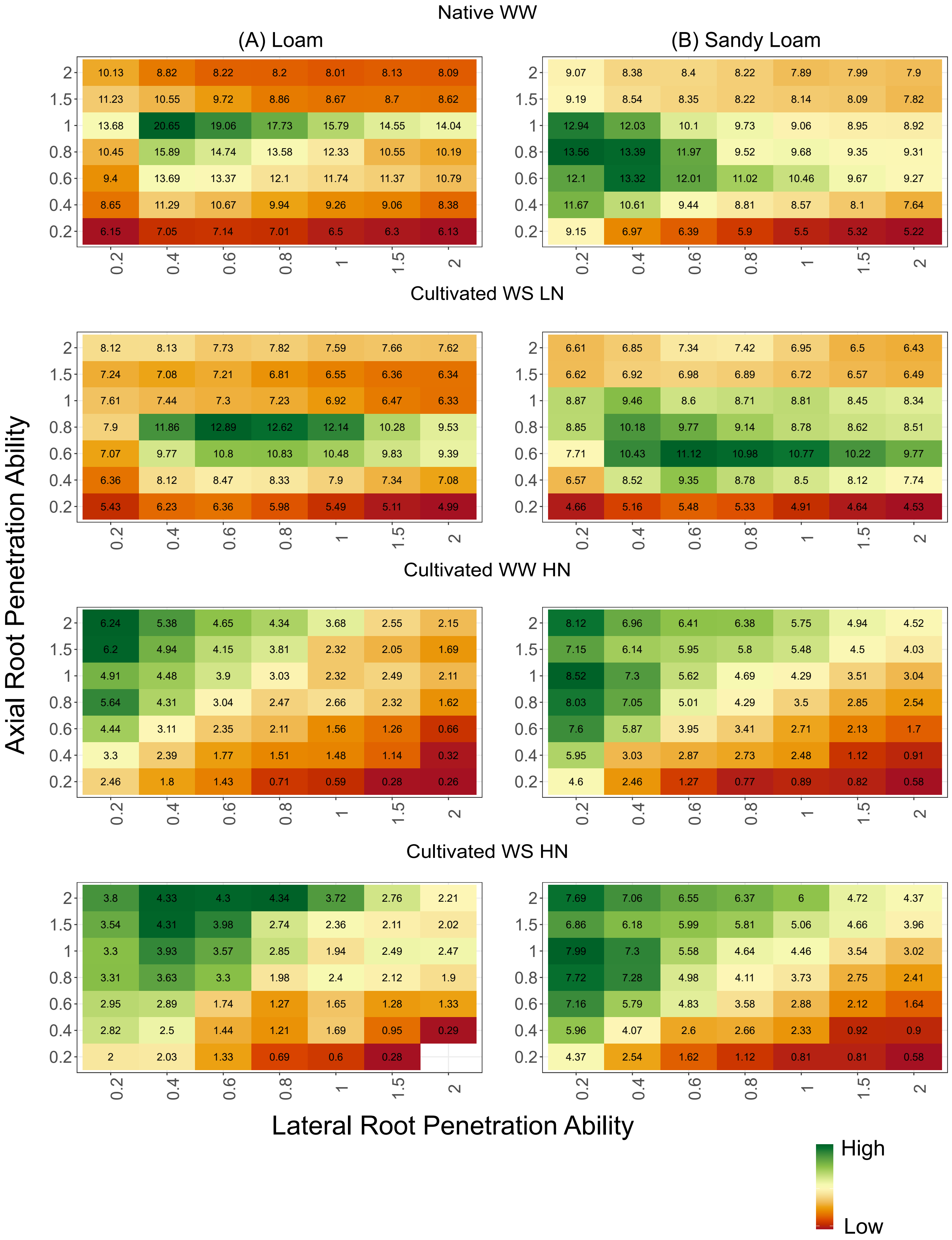
Native WW
(A) Loam
10.13 8.82 8.22 8.2 8.01 8.13 8.09
11.23 10.55 9.72 8.86 8.67 8.7 8.62
13.68 20.65 19.06 17.73 15.79 14.55 14.04
10.45 15.89 14.74 13.58 12.33 10.55 10.19
9.4 13.69 13.37 12.1 11.74 11.37 10.79
8.65 11.29 10.67 9.94 9.26 9.06 8.38
6.15 7.05 7.14 7.01 6.5 6.3 6.13
(B) Sandy Loam
9.07 8.38 8.4 8.22 7.89 7.99 7.9
9.19 8.54 8.35 8.22 8.14 8.09 7.82
12.94 12.03 10.1 9.73 9.06 8.95 8.92
13.56 13.39 11.97 9.52 9.68 9.35 9.31
12.1 13.32 12.01 11.02 10.46 9.67 9.27
11.67 10.61 9.44 8.81 8.57 8.1 7.64
9.15 6.97 6.39 5.9 5.5 5.32 5.22
Cultivated WS LN
8.12 8.13 7.73 7.82 7.59 7.66 7.62
7.24 7.08 7.21 6.81 6.55 6.36 6.34
7.61 7.44 7.3 7.23 6.92 6.47 6.33
7.9 11.86 12.89 12.62 12.14 10.28 9.53
7.07 9.77 10.8 10.83 10.48 9.83 9.39
6.36 8.12 8.47 8.33 7.9 7.34 7.08
5.43 6.23 6.36 5.98 5.49 5.11 4.99
6.61 6.85 7.34 7.42 6.95 6.5 6.43
6.62 6.92 6.98 6.89 6.72 6.57 6.49
8.87 9.46 8.6 8.71 8.81 8.45 8.34
8.85 10.18 9.77 9.14 8.78 8.62 8.51
7.71 10.43 11.12 10.98 10.77 10.22 9.77
6.57 8.52 9.35 8.78 8.5 8.12 7.74
4.66 5.16 5.48 5.33 4.91 4.64 4.53
Cultivated WW HN
6.24 5.38 4.65 4.34 3.68 2.55 2.15
6.2 4.94 4.15 3.81 2.32 2.05 1.69
4.91 4.48 3.9 3.03 2.32 2.49 2.11
5.64 4.31 3.04 2.47 2.66 2.32 1.62
4.44 3.11 2.35 2.11 1.56 1.26 0.66
3.3 2.39 1.77 1.51 1.48 1.14 0.32
2.46 1.8 1.43 0.71 0.59 0.28 0.26
8.12 6.96 6.41 6.38 5.75 4.94 4.52
7.15 6.14 5.95 5.8 5.48 4.5 4.03
8.52 7.3 5.62 4.69 4.29 3.51 3.04
8.03 7.05 5.01 4.29 3.5 2.85 2.54
7.6 5.87 3.95 3.41 2.71 2.13 1.7
5.95 3.03 2.87 2.73 2.48 1.12 0.91
4.6 2.46 1.27 0.77 0.89 0.82 0.58
Cultivated WS HN
3.8 4.33 4.3 4.34 3.72 2.76 2.21
3.54 4.31 3.98 2.74 2.36 2.11 2.02
3.3 3.93 3.57 2.85 1.94 2.49 2.47
3.31 3.63 3.3 1.98 2.4 2.12 1.9
2.95 2.89 1.74 1.27 1.65 1.28 1.33
2.82 2.5 1.44 1.21 1.69 0.95 0.29
2 2.03 1.33 0.69 0.6 0.28
7.69 7.06 6.55 6.37 6 4.72 4.37
6.86 6.18 5.99 5.81 5.06 4.66 3.96
7.99 7.3 5.58 4.64 4.46 3.54 3.02
7.72 7.28 4.98 4.11 3.73 2.75 2.41
7.16 5.79 4.83 3.58 2.88 2.12 1.64
5.96 4.07 2.6 2.66 2.33 0.92 0.9
4.37 2.54 1.62 1.12 0.81 0.81 0.58
2 1.5 1 0.8 0.6 0.4 0.2
0.2 0.4 0.6 0.8 1 1.5 2
Axial Root Penetration Ability
Lateral Root Penetration Ability
High
Low

Supplementary Data Fig. S11: Shoot biomass at 40 days after germination in phenotypes varying in axial and lateral root penetration ability in a loam (A) and sandy loam Cambisol (B) under four soil management scenarios as an irrigated cultivated soil with high N (Cultivated WW HN) , cultivated soil with dry topsoil and high N (Cultivated WS HN), well-watered native soil (Native WW) , cultivated soil with dry topsoil and low N (Cultivated WS LN) in a low CO_2 environment. The maize root phenotypes vary in axial and lateral root penetration ability.

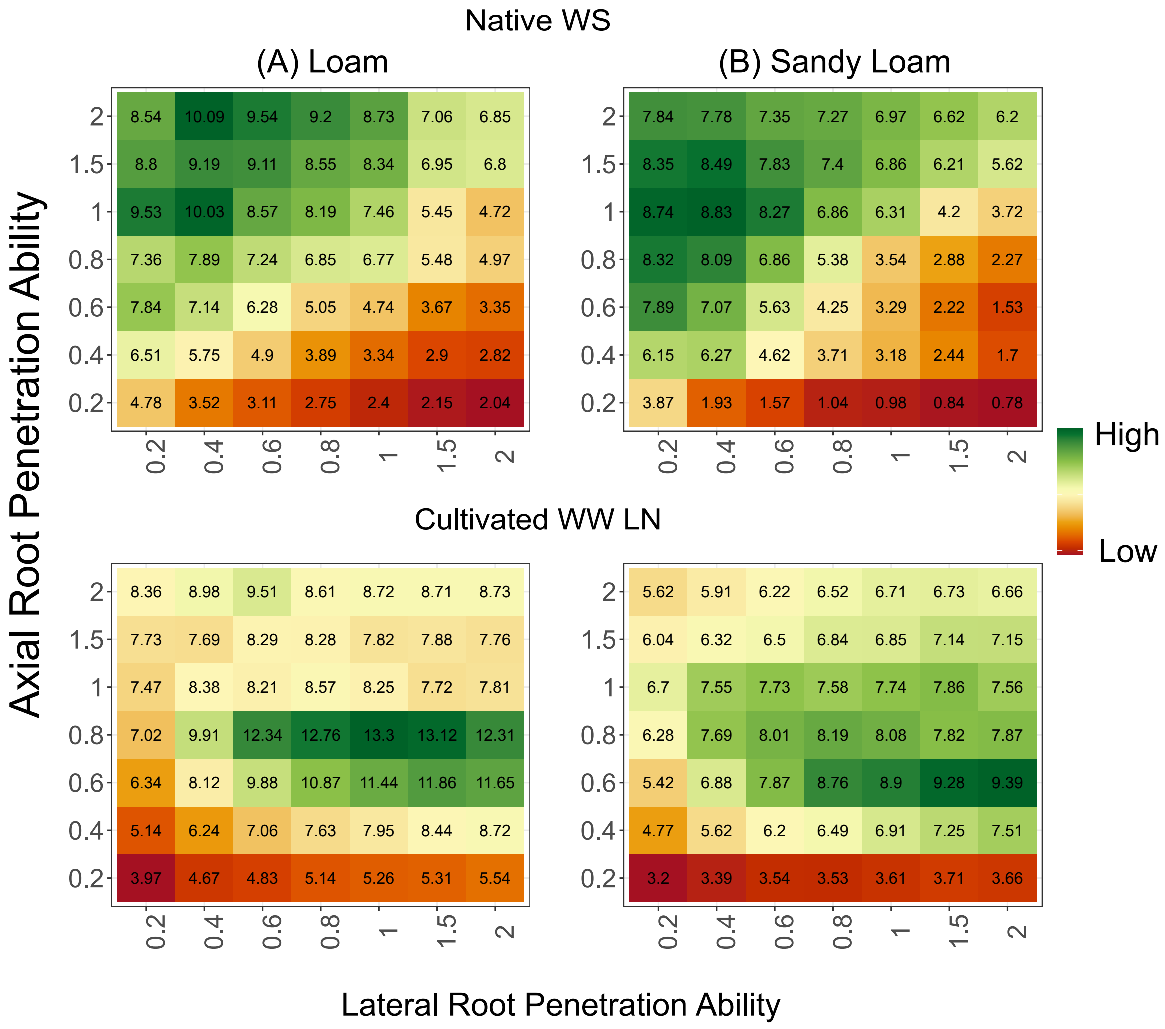

Supplementary Data Fig. S12. Shoot biomass at 40 days after germination in maize phenotypes varying in axial and lateral root penetration ability in a loam and sandy loam Cambisol as a native undisturbed soil with dry topsoil (Native WS) and an irrigated cultivated soil with low N (Cultivated WW LN) respectively in an environment with 500 ppm CO_2.

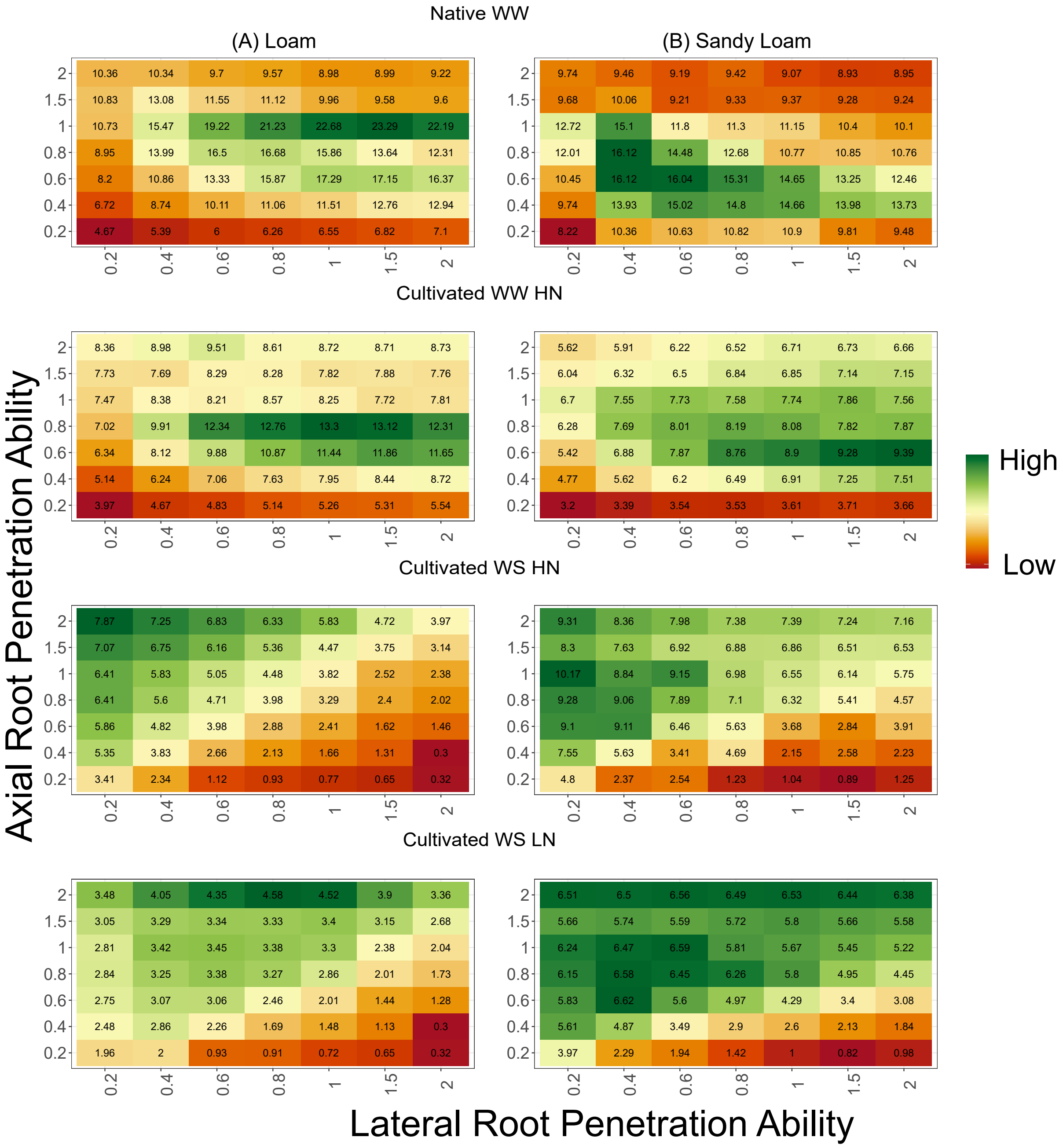
Native WW
(A) Loam
(B) Sandy Loam
Cultivated WW HN
Cultivated WS HN
Cultivated WS LN
Axial Root Penetration Ability
Lateral Root Penetration Ability
High
Low

Supplementary Data Fig. S13: Shoot biomass at 40 days after germination in phenotypes varying in axial and lateral root penetration ability in a loam (A) and sandy loam Cambisol (B) as an irrigated cultivated soil with high N (Cultivated WW HN) , cultivated soil with dry topsoil and high N (Cultivated WS HN), well-watered native soil (Native WW) , cultivated soil with dry topsoil and low N (Cultivated WS LN) in a high CO_2 environment (500 ppm).

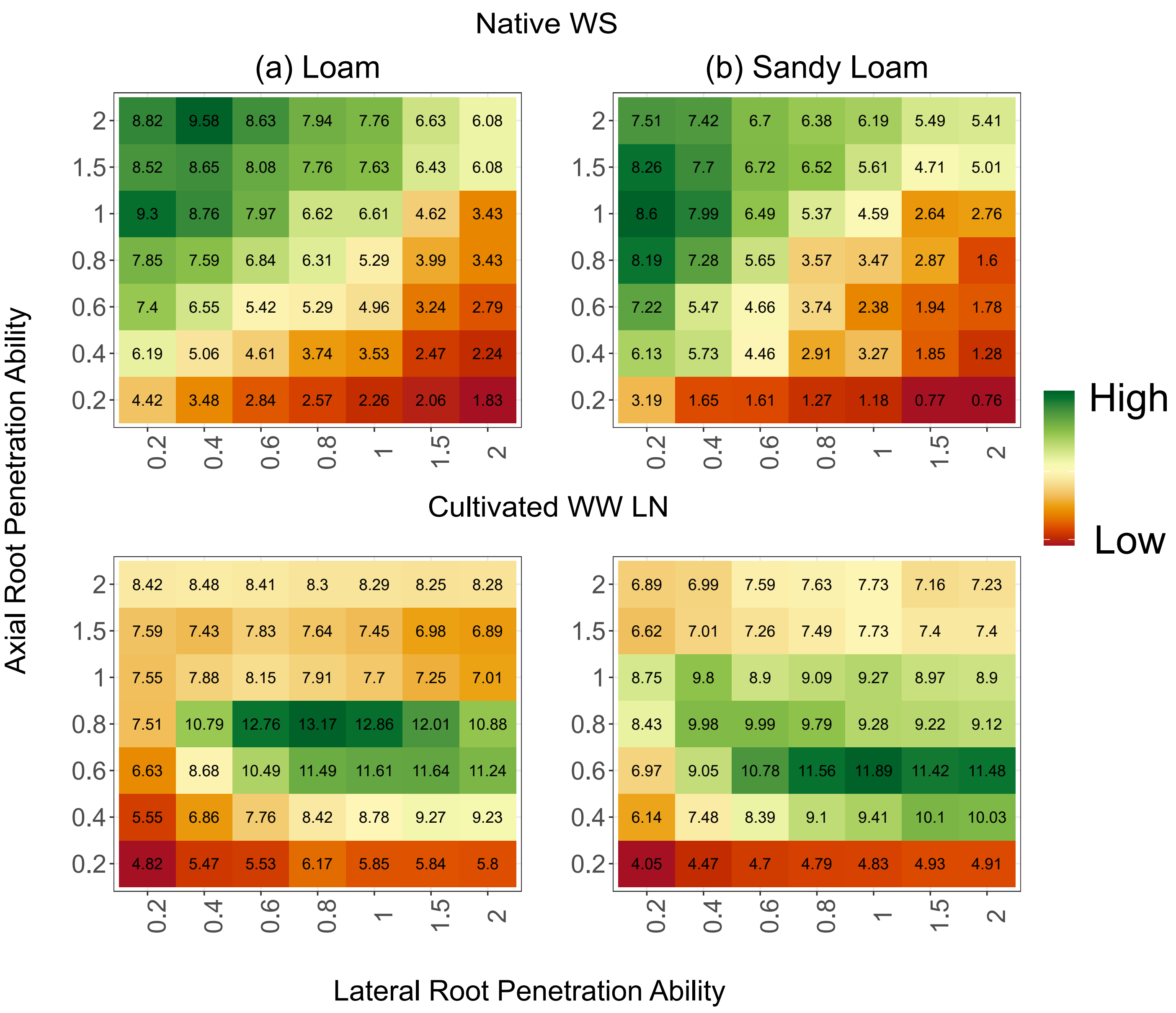

Supplementary Data Fig. S14. Shoot biomass at 40 days after germination in maize phenotypes varying in axial and lateral root penetration ability in a loam and sandy loam Cambisol as a native undisturbed soil with dry topsoil (Native WS) and an irrigated cultivated soil with low N (Cultivated WW LN) respectively in an environment with 402.9 ppm CO_2.

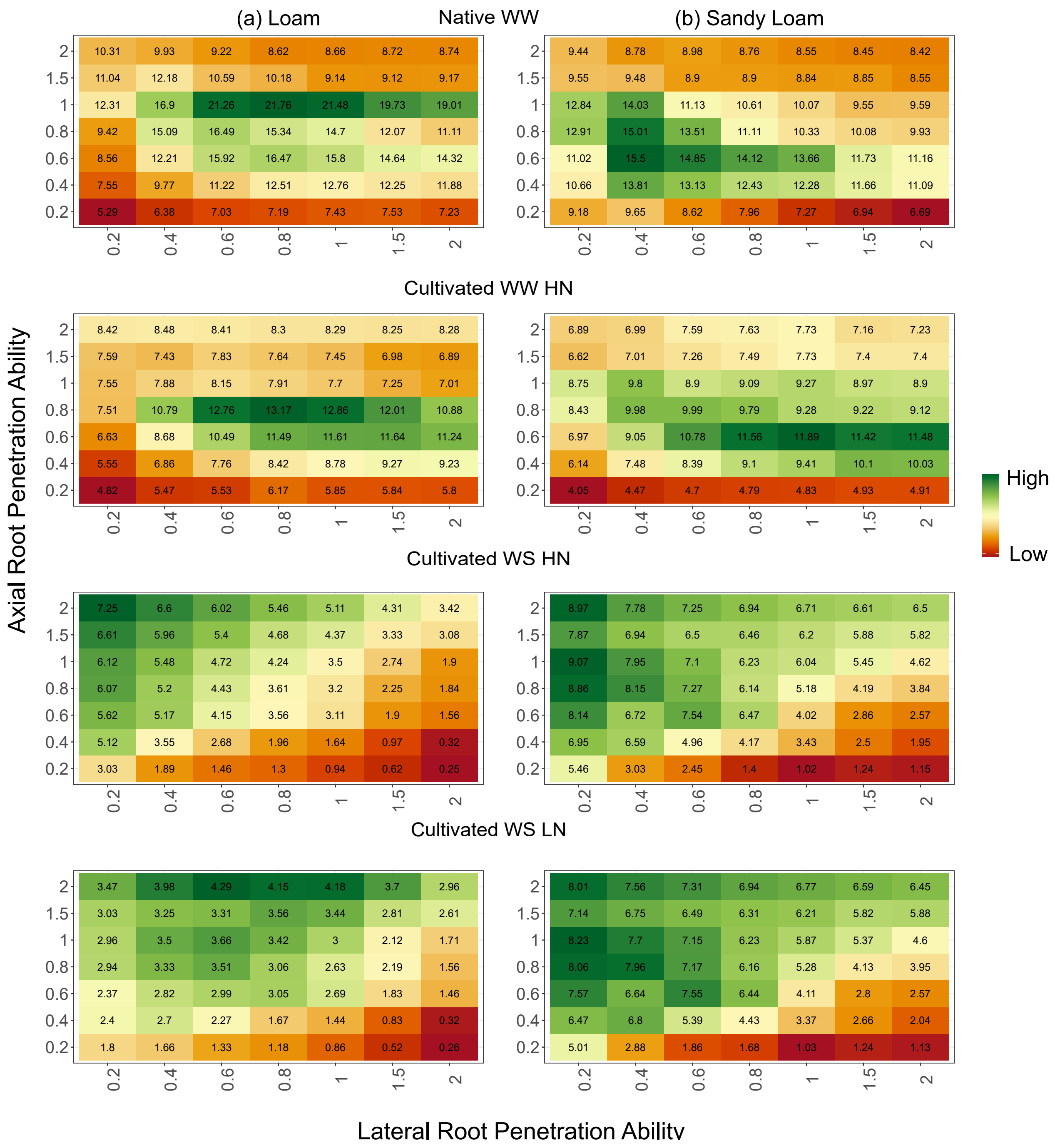
(a) Loam
Native WW
(b) Sandy Loam
2 10.31 9.93 9.22 8.62 8.66 8.72 8.74
1.5 11.04 12.18 10.59 10.18 9.14 9.12 9.17
1 12.31 16.9 21.26 21.76 21.48 19.73 19.01
0.8 9.42 15.09 16.49 15.34 14.7 12.07 11.11
0.6 8.56 12.21 15.92 16.47 15.8 14.64 14.32
0.4 7.55 9.77 11.22 12.51 12.76 12.25 11.88
0.2 5.29 6.38 7.03 7.19 7.43 7.53 7.23
0.2 0.4 0.6 0.8 1 1.5 2
2 9.44 8.78 8.98 8.76 8.55 8.45 8.42
1.5 9.55 9.48 8.9 8.9 8.84 8.85 8.55
1 12.84 14.03 11.13 10.61 10.07 9.55 9.59
0.8 12.91 15.01 13.51 11.11 10.33 10.08 9.93
0.6 11.02 15.5 14.85 14.12 13.66 11.73 11.16
0.4 10.66 13.81 13.13 12.43 12.28 11.66 11.09
0.2 9.18 9.65 8.62 7.96 7.27 6.94 6.69
0.2 0.4 0.6 0.8 1 1.5 2
Cultivated WW HN
2 8.42 8.48 8.41 8.3 8.29 8.25 8.28
1.5 7.59 7.43 7.83 7.64 7.45 6.98 6.89
1 7.55 7.88 8.15 7.91 7.7 7.25 7.01
0.8 7.51 10.79 12.76 13.17 12.86 12.01 10.88
0.6 6.63 8.68 10.49 11.49 11.61 11.64 11.24
0.4 5.55 6.86 7.76 8.42 8.78 9.27 9.23
0.2 4.82 5.47 5.53 6.17 5.85 5.84 5.8
0.2 0.4 0.6 0.8 1 1.5 2
2 6.89 6.99 7.59 7.63 7.73 7.16 7.23
1.5 6.62 7.01 7.26 7.49 7.73 7.4 7.4
1 8.75 9.8 8.9 9.09 9.27 8.97 8.9
0.8 8.43 9.98 9.99 9.79 9.28 9.22 9.12
0.6 6.97 9.05 10.78 11.56 11.89 11.42 11.48
0.4 6.14 7.48 8.39 9.1 9.41 10.1 10.03
0.2 4.05 4.47 4.7 4.79 4.83 4.93 4.91
0.2 0.4 0.6 0.8 1 1.5 2
Axial Root Penetration Ability
High
Low
Cultivated WS HN
2 7.25 6.6 6.02 5.46 5.11 4.31 3.42
1.5 6.61 5.96 5.4 4.68 4.37 3.33 3.08
1 6.12 5.48 4.72 4.24 3.5 2.74 1.9
0.8 6.07 5.2 4.43 3.61 3.2 2.25 1.84
0.6 5.62 5.17 4.15 3.56 3.11 1.9 1.56
0.4 5.12 3.55 2.68 1.96 1.64 0.97 0.32
0.2 3.03 1.89 1.46 1.3 0.94 0.62 0.25
0.2 0.4 0.6 0.8 1 1.5 2
2 8.97 7.78 7.25 6.94 6.71 6.61 6.5
1.5 7.87 6.94 6.5 6.46 6.2 5.88 5.82
1 9.07 7.95 7.1 6.23 6.04 5.45 4.62
0.8 8.86 8.15 7.27 6.14 5.18 4.19 3.84
0.6 8.14 6.72 7.54 6.47 4.02 2.86 2.57
0.4 6.95 6.59 4.96 4.17 3.43 2.5 1.95
0.2 5.46 3.03 2.45 1.4 1.02 1.24 1.15
0.2 0.4 0.6 0.8 1 1.5 2
Cultivated WS LN
2 3.47 3.98 4.29 4.15 4.18 3.7 2.96
1.5 3.03 3.25 3.31 3.56 3.44 2.81 2.61
1 2.96 3.5 3.66 3.42 3 2.12 1.71
0.8 2.94 3.33 3.51 3.06 2.63 2.19 1.56
0.6 2.37 2.82 2.99 3.05 2.69 1.83 1.46
0.4 2.4 2.7 2.27 1.67 1.44 0.83 0.32
0.2 1.8 1.66 1.33 1.18 0.86 0.52 0.26
0.2 0.4 0.6 0.8 1 1.5 2
2 8.01 7.56 7.31 6.94 6.77 6.59 6.45
1.5 7.14 6.75 6.49 6.31 6.21 5.82 5.88
1 8.23 7.7 7.15 6.23 5.87 5.37 4.6
0.8 8.06 7.96 7.17 6.16 5.28 4.13 3.95
0.6 7.57 6.64 7.55 6.44 4.11 2.8 2.57
0.4 6.47 6.8 5.39 4.43 3.37 2.66 2.04
0.2 5.01 2.88 1.86 1.68 1.03 1.24 1.13
0.2 0.4 0.6 0.8 1 1.5 2
Lateral Root Penetration Abilitv

Supplementary Data Fig. S15: Shoot biomass at 40 days after germination in phenotypes varying in axial and lateral root penetration ability in a loam (A) and sandy loam Cambisol (B) under four soil management scenarios as an irrigated cultivated soil with high N (Cultivated WW HN) , cultivated soil with dry topsoil and high N (Cultivated WS HN), well-watered native soil (Native WW) , cultivated soil with dry topsoil and low N (Cultivated WS LN) in an environment with 402.9 ppm CO_2.
