# Supplementary material for: Did crop domestication change the fitness landscape of root response to soil mechanical impedance? An in silico analysis
Source: Ann Bot. 2024 Nov 27;136(5-6):997–1011. doi: 10.1093/aob/mcae201 (PMC12682825; doi:10.1093/aob/mcae201)
Supplement: mcae201_suppl_Supplementary_Table_S1 [file mcae201_suppl_supplementary_table_s1.docx]

| Depth  (cm) | θr  _cm_^3^ _cm_^-3^ | θs  _cm_^3^ _cm_^-3^ | α  _(hPa_^-1^_)_ | n | K_sat_  _(cm day_ ^-1^_)_ | D_b_  _(g cm_ ^-3^_)_ | Org.Matter | Soil | Soil.Management |
| --- | --- | --- | --- | --- | --- | --- | --- | --- | --- |
| 0 | 0.0353 | 0.4155 | 0.0133 | 1.3871 | 38.48 | 1.43 | 1.13 | Loam | Native |
| -22 | 0.0803 | 0.5097 | 0.0076 | 1.2432 | 4.38 | 1.45 | 0.41 | Loam | Native |
| -47 | 0.0766 | 0.4957 | 0.0061 | 1.2755 | 4.32 | 1.5 | 0.34 | Loam | Native |
| -66 | 0.0723 | 0.4845 | 0.0051 | 1.3023 | 4.46 | 1.56 | 0.35 | Loam | Native |
| -89 | 0.0543 | 0.4209 | 0.0063 | 1.3876 | 9.23 | 1.6 | 0.25 | Loam | Native |
| -123 | 0.0413 | 0.3881 | 0.0316 | 1.3346 | 46.26 | 1.53 | 0.23 | Loam | Native |
| -161 | 0.0312 | 0.3978 | 0.0569 | 1.5602 | 156.9 | 1.46 | 0.2 | Loam | Native |
| -200 | 0.0312 | 0.3978 | 0.0569 | 1.5602 | 156.9 | 1.57 | 0.22 | Loam | Native |
| 0 | 0.0349 | 0.4142 | 0.0114 | 1.3882 | 38.04 | 1.573 | 0.565 | Loam | Cultivated |
| -11 | 0.0911 | 0.5097 | 0.0108 | 1.2495 | 4.38 | 1.45 | 0.41 | Loam | Cultivated |
| -36 | 0.0873 | 0.4957 | 0.0088 | 1.2822 | 4.32 | 1.5 | 0.34 | Loam | Cultivated |
| -55 | 0.084 | 0.4845 | 0.0077 | 1.3105 | 4.46 | 1.56 | 0.35 | Loam | Cultivated |
| -78 | 0.0562 | 0.4209 | 0.007 | 1.3913 | 9.23 | 1.6 | 0.25 | Loam | Cultivated |
| -112 | 0.0413 | 0.3881 | 0.0316 | 1.3346 | 46.26 | 1.53 | 0.23 | Loam | Cultivated |
| -151 | 0.0312 | 0.3978 | 0.0569 | 1.5602 | 156.9 | 1.46 | 0.2 | Loam | Cultivated |
| -200 | 0.0312 | 0.3978 | 0.0569 | 1.5602 | 156.9 | 1.57 | 0.22 | Loam | Cultivated |
| 0 | 0.0249 | 0.4244 | 0.0429 | 1.3834 | 141.6 | 1.46 | 0.82 | Sandy Loam | Native |
| -26 | 0.0344 | 0.4027 | 0.0469 | 1.3834 | 84 | 1.36 | 0.45 | Sandy Loam | Native |
| -47 | 0.0352 | 0.397 | 0.0493 | 1.4171 | 96 | 1.48 | 0.29 | Sandy Loam | Native |
| -73 | 0.0617 | 0.4168 | 0.0166 | 1.3106 | 12 | 1.42 | 0.3 | Sandy Loam | Native |
| -97 | 0.0513 | 0.4068 | 0.0161 | 1.3312 | 19.2 | 1.48 | 0.25 | Sandy Loam | Native |
| -115 | 0.0432 | 0.3922 | 0.0339 | 1.3459 | 48 | 1.53 | 0.21 | Sandy Loam | Native |
| -130 | 0.0311 | 0.4007 | 0.0611 | 1.5812 | 158.4 | 1.46 | 0.16 | Sandy Loam | Native |
| -170 | 0.0311 | 0.4007 | 0.0611 | 1.5812 | 158.4 | 1.57 | 0.14 | Sandy Loam | Native |
| 0 | 0.0236 | 0.4054 | 0.0409 | 1.3869 | 131.27 | 1.606 | 0.41 | Sandy Loam | Cultivated |
| -13 | 0.0331 | 0.3933 | 0.0383 | 1.3693 | 79.86 | 1.36 | 0.225 | Sandy Loam | Cultivated |
| -34 | 0.0342 | 0.3912 | 0.0452 | 1.4036 | 92.58 | 1.48 | 0.145 | Sandy Loam | Cultivated |
| -60 | 0.0563 | 0.4115 | 0.0138 | 1.2995 | 11.58 | 1.42 | 0.15 | Sandy Loam | Cultivated |
| -84 | 0.0483 | 0.4017 | 0.0143 | 1.3222 | 17.82 | 1.48 | 0.125 | Sandy Loam | Cultivated |
| -102 | 0.0413 | 0.3881 | 0.0316 | 1.3346 | 46.26 | 1.53 | 0.105 | Sandy Loam | Cultivated |
| -117 | 0.0312 | 0.3978 | 0.0569 | 1.5602 | 156.9 | 1.46 | 0.08 | Sandy Loam | Cultivated |
| -157 | 0.0312 | 0.3978 | 0.0569 | 1.5602 | 156.9 | 1.57 | 0.07 | Sandy Loam | Cultivated |

Supplementary Data Table. S1: Bulk density D_b_ and the van Genutchen parameters θr (residual water content), θs (saturated water content), α, n and the saturated hydraulic conductivity K_s_ for the loam and sandy loam Inceptisol. α and n describe the shape of the soil-water retention curve.
